# Ramatroban-Based Analogues Containing Fluorine Group as Potential ^18^F-Labeled Positron Emission Tomography (PET) G-Protein Coupled Receptor 44 (GPR44) Tracers

**DOI:** 10.3390/molecules26051433

**Published:** 2021-03-06

**Authors:** Lina A. Huang, Kelly X. Huang, Jui Tu, Fouad Kandeel, Junfeng Li

**Affiliations:** Department of Translational Research & Cellular Therapeutics, Beckman Research Institute of the City of Hope, Duarte, CA 91010, USA; lahuang@exeter.edu (L.A.H.); kellyhuang1220@g.ucla.edu (K.X.H.); jutu@coh.org (J.T.); FKandeel@coh.org (F.K.)

**Keywords:** positron emission tomography (PET), G-protein coupled receptor 44 (GPR44), CRTH2, diabetes, beta cell mass (BCM), pancreatic islets, beta cell imaging, ^18^F-labeling, ramatroban analogues, PET probes

## Abstract

Diabetes remains one of the fastest growing chronic diseases and is a leading source of morbidity and accelerated mortality in the world. Loss of beta cell mass (BCM) and decreased sensitivity to insulin underlie diabetes pathogenesis. Yet, the ability to safely and directly assess BCM in individuals with diabetes does not exist. Measures such as blood glucose provide only a crude indirect picture of beta cell health. PET imaging could, in theory, allow for safe, direct, and precise characterization of BCM. However, identification of beta cell-specific radiolabeled tracers remains elusive. G-protein coupled receptor 44 (GPR44) is a transmembrane protein that was characterized in 2012 as highly beta cell-specific within the insulin-positive islets of Langerhans. Accordingly, radiolabeling of existing GPR44 antagonists could be a viable method to accelerate PET tracer development. The present study aims to evaluate and summarize published analogues of the GPR44 antagonist ramatroban to develop ^18^F-labeled PET tracers for BCM analysis. The 77 corresponding ramatroban analogues containing a fluorine nuclide were characterized for properties including binding affinity, selectivity, and pharmacokinetic and metabolic profile, and 32 compounds with favorable properties were identified. This review illustrates the potential of GPR44 analogues for the development of PET tracers.

## 1. Introduction

### 1.1. Positron Emission Tomography (PET) Imaging for Beta Cell Mass (BCM)

Diabetes is a chronic disease characterized by the disruption of glucose homeostasis and a major cause of limb amputation, stroke, renal failure, and heart disease. Diabetes affected approximately 476 million people globally in 2017 and has become the seventh leading cause of death [1]. Type 1 diabetes (T1D) and type 2 diabetes (T2D) are two primary diagnoses, and pathology of both is caused by insulin dysregulation and subsequent hyperglycemia. In both T1D and T2D, the pathogenesis and severity of disease can be elucidated from beta cell mass (BCM). Access to BCM measurements in vivo is pivotal for determining the progression of BCM during disease pathogenesis and monitoring effects of diabetes treatments. However, post-mortem biopsies are currently the primary method to study BCM [2,3]. Routine biopsy sampling of living patients is restricted by the relatively inaccessible location of the pancreas in the body and the invasive nature of the procedure. In fact, a clinical study of T1D patients showed significant complications associated with the procedure, including bleeding and pancreatic leakage [4]. Another approach utilizing plasma markers, such as insulin, c-peptide, and glycated hemoglobin (HbA1c), fails to measure BCM accurately. The downstream metabolic processes which form these markers may hamper their correlation to BCM. These markers have also been shown to strongly fluctuate in association with metabolism [5]. As such, current methods do not allow for accurate and non-invasive in vivo longitudinal evaluation of BCM progression [3].

To overcome the limitations of current approaches to BCM analysis, positron emission tomography (PET) has been explored as a viable alternative. PET is a non-invasive, quantitative technique that assesses radioactive tracer concentrations in vivo. Since the tracer is presumably bound to a receptor, the receptor location and density can be mapped through computer analysis of the gamma rays. As such, PET imaging of BCM has the potential to follow the progression of metabolic diseases, support beta cell replacement technologies, and assist pharmaceutical drug development. The efficacy of PET imaging, however, depends in large part on the tracer selected, particularly because beta cells make up only 1% of the pancreas mass [6]. Accordingly, the application of PET to BCM analysis depends upon the development of a highly specific radiotracer targeting beta cells. While several cell-surface molecules, such as VMAT2 and GLP-1R, have been proposed, they have been found lacking in specificity [7,8]. Therefore, we are interested in identifying and assessing novel beta cell-specific molecular targets for PET tracer development.

### 1.2. G-Protein Coupled Receptor 44 (GPR44) and Related PET Tracers

In 1999, Marchese et al. conducted a PCR study to isolate novel G-protein coupled receptors (GPCR) based on oligonucleotide primers [9,10]. One of the GPCRs they identified was GPR44, a 7-helix transmembrane protein. In later studies, GPR44 was also referred to as prostaglandin D_2_ receptor 2 (DP_2_) and chemoattractant receptor-homologous molecule expressed on T helper type 2 cells (CRTH_2_). GPR44 was then applied for the investigation and treatment of metabolic and inflammatory diseases, and showed promising results. In 2012, the first study characterizing the expression of GPR44 in human islets used immunofluorescent staining to reveal that GPR44 is highly beta cell-specific, contained within the insulin-positive islets of Langerhans, and non-specific for glucagon [11]. Further studies continued to indicate that GPR44 could serve as a viable marker of BCM [12,13].

**Figure 1:**
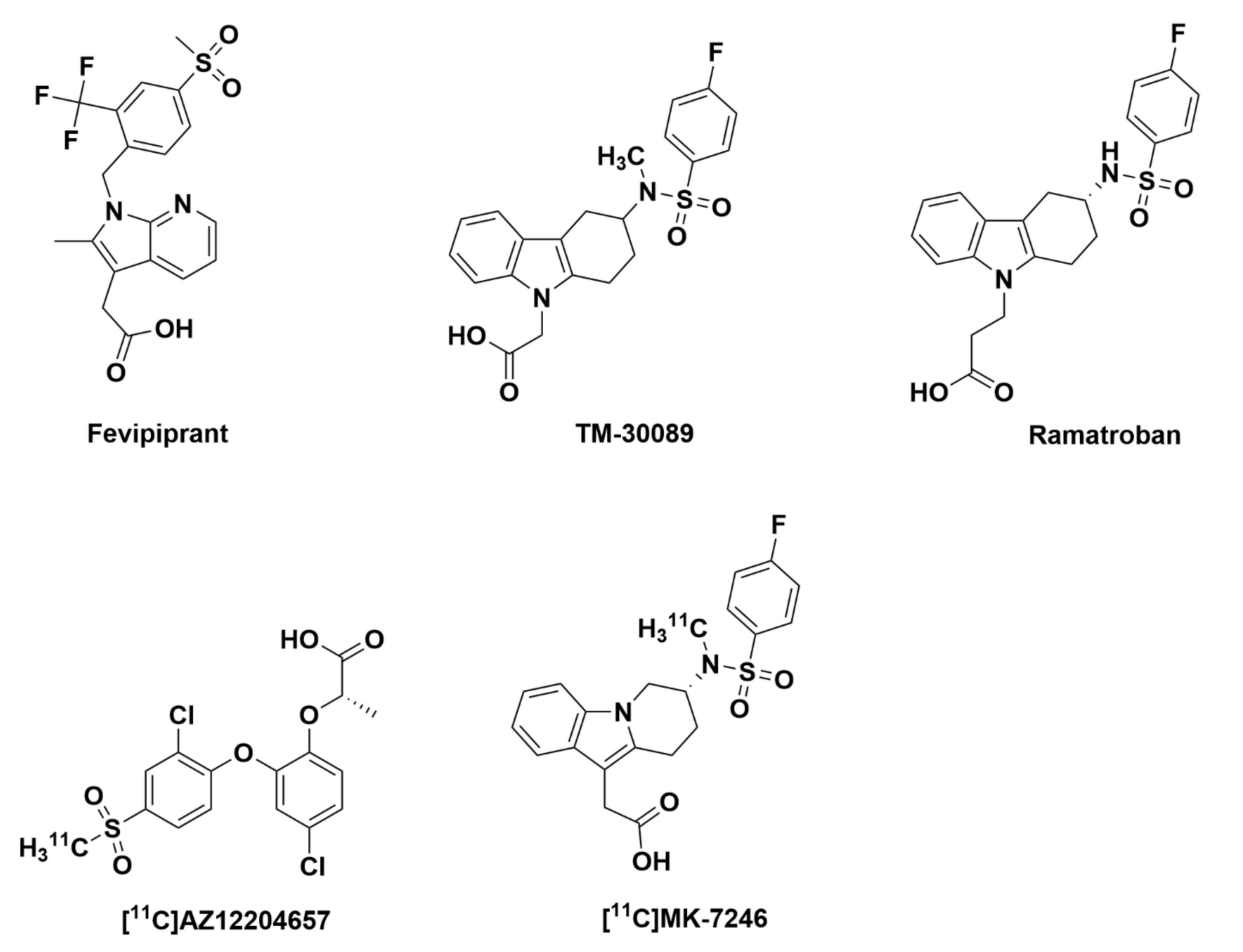
Molecular structures of **fevipiprant, TM-30089, ramatroban, [^11^C]AZ12204657** and **[^11^C]MK-7246**.

In 2001, binding assays revealed that the GPR44 agonist was pro-inflammatory mediator prostaglandin D_2_ (PGD_2_) [14,15]. PGD_2_ is a lipid molecule that has been implicated in asthma, allergic rhinitis, and other diseases involving type 2 inflammation. PGD_2_ binding to GPR44 causes an increase in cytoplasmic levels of Ca^2+^, which inhibits cAMP production and subsequently induces cell migration and activation [16]. As might be expected from their common binding to GPR44, PGD_2_ and other GPR44 ligands share many structural similarities. For instance, most GPR44 antagonists contain a carboxylic acid-derived region that is likely critical for binding [17]. An in silico study characterizing the binding mechanisms between GPR44 and two of its antagonists (Figure 1), **fevipiprant** (2-[2-methyl-1-[[4-methylsulfonyl-2-(trifluoromethyl)phenyl]methyl]pyrrolo[2,3-b]pyridin-3-yl]acetic acid) and **TM-30089** ({3-[(4-fluoro-benzenesulfonyl)methyl-amino]-1,2,3,4-tetrahydro-carbazol-9-yl}acetic acid), (to be discussed in more detail in Section 2.2.2.), showed that the carboxylate group of the antagonist is possibly attracted to GPR44’s highly positively charged surface. The antagonist then enters the receptor through a gap between transmembrane helix 1 (TM1) and transmembrane helix 7 (TM7). Upon entering, the antagonist binds to a site that contains mostly aromatic residues, which matches well with the polar acetate group attached to a central aromatic group found in many GPR44 antagonists [18].

There exist a number of known and developed GPR44 ligands, but only two have been characterized as potential GPR44 PET tracers in Figure 1: **[^11^C]AZ12204657** ([^11^C]- ((*S*)-2-(4-chloro-2-[2-chloro-4-(methylsulfonyl)phenoxy]phenoxy) propanoic acid) and **[^11^C]MK-7246** ([^11^C]-{(7*R*)-7-[[(4-fluorophenyl)sulfonyl](methyl)amino]-6,7,8,9-tetrahydropyrido[1,2-a]indol-10-yl}acetic acid). Interest in these ligands began when GPR44 antagonists **AZ12204657** (IC_50_ = 2.5 nM and K_d_ = 2.9 µg), a structural analogue of the high-affinity GPR44 antagonist **AZD3825**, and **MK-7246** ({(7*R*)-7-[[(4-fluorophenyl)sulfonyl](methyl)amino]-6,7,8,9-tetrahydropyrido[1,2-a]indol-10-yl}acetic acid), a derivative of **TM-30089**, demonstrated specific binding to insulin-positive islets in human pancreatic sections [12,19,20]. These compounds were subsequently radiolabeled with carbon-11 and investigated as PET tracers in animal models. While **MK-7246** will be discussed further in Section 2.2.3, it is worth noting that **AZ12204657** has been developed into a PET tracer for BCM. In a mouse model with kidney capsule-engrafted human islets, **[^11^C]AZ12204657** was shown to strongly bind to the capsule containing the transplants but expressed negligible binding to the non-transplanted kidney capsule [12,19]. This binding was confirmed to be GPR44-mediated, as it could be inhibited by pre-treatment with **AZD3825**. In addition, **[^11^C]AZ12204657** demonstrated focal binding patterns consistent with insulin-positive areas in the pancreas of non-diabetic pigs.

However, the tracers studied insofar have been mainly labeled with carbon-11, which limits the application of these probes to a short half-life of 20 min and a viability of no longer than 2 h, after which radioactive imaging properties are lost. The application of ^11^C-labeling is further limited because PET tracers generally are suitable for clinical use within three half-lives, meaning that radiotracer quality testing should be set to be within 1 h [21]. Additionally, radiosynthesis of these probes requires artificial production of carbon-11 by an on-site cyclotron. These limitations do not allow tracers to be administered to multiple patients, delivered to remote sites, or implemented in preclinical validation assays.

Due to the limitations presented by ^11^C-labeled tracers, ^18^F-labeling is a more suitable and effective alternative for development of GPR44 PET tracers, as fluorine-18 has a half-life of 109.7 min that allows for up to 10 h of imaging. Additionally, fluorine-18 has a 97% positron emission (β+ decay), low maximum positron energy (0.635 MeV), favorable van der Waals radius (1.47 Å), and high electronegativity, with the corresponding high-energy bond with carbon (112 kcal/mol). The bonding with carbon is important, as it lends the radiolabeled tracer stability in high temperatures and oxidation resistance. As opposed to another prominent radioisotope, gallium-68, the low positron energy of fluorine-18 results in a short diffusion range (< 2.4 mm) for optimal imaging resolution [22]. Although the spatial resolution, sensitivity, contrast, and activity recovery coefficients do fluctuate depending on the PET tracer used, a comparison of fluorine-18 and gallium-68 imaging found that PET scanners performed better with fluorine-18, particularly high-resolution small animal PET scanners [23]. Currently, most of the PET agents used in clinical studies are ^18^F-labeled. 

Development of ^18^F-labeled GPR44 PET tracers is of interest due to the positive results shown by these previous studies. Approaches to do so include modifying previously existing GPR44 antagonists and clinically evaluated drugs. In particular, the benefit of the drug approach is that the molecular structures and properties of such compounds have already been studied, and the appendage of the molecule may be simply replaced with a radioactive moiety to serve as a PET tracer.

The present paper aims to summarize current **ramatroban**-based GPR44 ligands containing a fluorine group and evaluate their potential as targets for the development of novel ^18^F-labeled PET tracers. An important consideration for ^18^F-labeling is that the potential molecule must already contain a fluorine group in the chemical structure to allow for ^18^F-labeling of the structures without significantly altering the pharmacological property of the molecule. 

## 2. Ramatroban-Based Analogues for Potential GPR44 Binding

### 2.1. Ramatroban

In 1989, Rosentreter et al. at Bayer AG synthesized the novel indole sulfonamide, **ramatroban** ((3*R*)-3-(4-Fluorophenylsulfonamido)-1,2,3,4-tetrahydro-9-carbazolepropanoic acid). **Ramatroban** (Figure 1), also known as **BAY u 3405**, was first described as an antagonist of thromboxane receptor (TP) and later shown to also serve as an antagonist to GPR44. Initially, **ramatroban** was found to bind to TP after displacing the TP antagonist **[^3^H]SQ29548** in a binding assay, resulting in an IC_50_ of 68 nM [24]. Additional binding assays with TP agonist **U-46619** [25] showed stronger antagonist binding to TP, with a lower IC_50_ of 30 nM and K_i_ of 10 nM [26]. The K_d_ and B_max_ of **ramatroban** were characterized as around 6 nM and 1177 binding sites/platelet, respectively [27].

Due to its binding to TP, **ramatroban** was developed for TP-mediated cardiovascular disorders. Specifically, **ramatroban** was found to inhibit platelet aggregation and deposition [28,29,30,31,32,33,34], contraction of smooth muscle [35,36,37], sudden death [38,39,40], myocardial infarctions [38,41,42,43], splenic artery occlusion shock [44], acute ischemia-reperfusion injury [42], and thrombosis [29,45,46,47,48,49]. In 2014, a study of constitutively active mutants (CAMs) in TP demonstrated **ramatroban** binding, in which **ramatroban** reduced basal activity [50]. In binding to TP, **ramatroban** targets TM1, and the carboxylic acid group of **ramatroban** forms polar attractions to TM2 and TM7 [51].

In vitro assays showed that **ramatroban** selectively inhibited PGD_2_-mediated responses. Displacement of TP agonists **U-46619** and I-BOP demonstrated that **ramatroban** acts competitively on TP [52]. In this method, PGD_2_ concentration curves shifted to the right by a pA_2_ of 7–8.5 after **ramatroban** introduction, characteristic of a competitive inhibitors [53,54]. **Ramatroban** inhibition of PGD_2_, which binds to both TP and GPR44, led to the later discovery that **ramatroban** serves as a dual antagonist for TP and GPR44. Further confirmation was provided through investigations on PGD_2_-mediated mechanisms, including eosinophil shape change assays, and in vivo displacement [55].

However, **ramatroban** binding to GPR44 was about 16 times less selective than for TP. This binding affinity for GPR44 was elucidated in human embryonic kidney cells (HEK293 cells) transfected with GPR44, and the K_i_ value was found to be 290 nM [56]. The **ramatroban** IC_50_ value for GPR44 in various assays proved comparably weak at 100 nM [53,57]. Additionally, **ramatroban** exhibited a half-life in binding assays of 4.60 min [55]. The influence of **ramatroban** inhibition on GPR44 is particularly evident in studies of eosinophils, basophils, and T helper type 2 (TH2) cells migration or count [58,59,60,61]. In vitro flow cytometry found that **ramatroban** could inhibit eosinophil and basophil shape change [62,63], and inhibit mast cell-induced TH2 migration in vitro [64].

Overall, the poor selectivity for GPR44 (unacceptable dual binding to TP and GPR44) suggests **ramatroban** may not be optimal as a PET tracer for measuring BCM. However, to date, numerous compounds have been derived from **ramatroban** that suggests further directions for developing GPR44-specific PET tracers. Described below are fluorine nuclide-containing **ramatroban** analogues, which we defined as molecules either explicitly stated as **ramatroban** derivatives in the papers or molecules that contain the characteristic tetrahydrocarbazole group of **ramatroban**.

### 2.2. Ramatroban Analogues

#### 2.2.1. Athersys, Inc.

**Figure 2:**
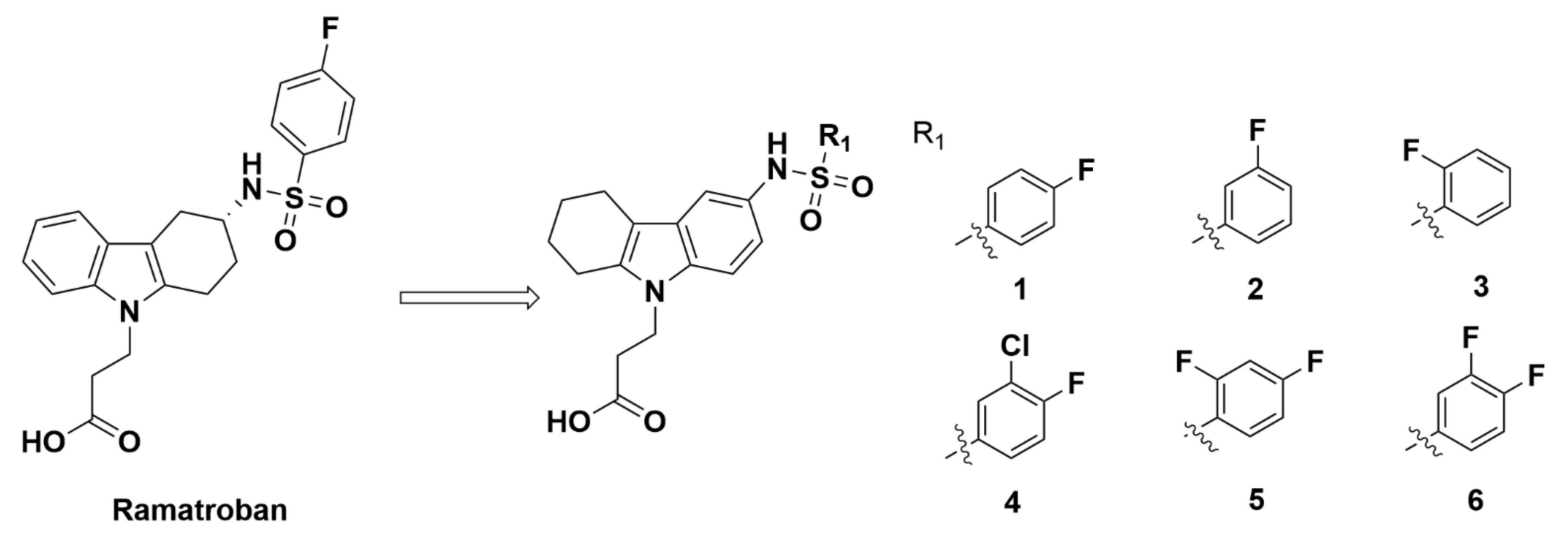
Molecular structures of compounds **1**–**6**.

In 2005, after **ramatroban** was identified as a GPR44 antagonist, Robarge et al. at Athersys, Inc. synthesized several **ramatroban** analogues to elucidate pharmacophores with promising affinity for GPR44. The analogues were created by changing the substituents and/or the location of the substitution [56], in which the scaffold of benzenesulfonamide was inserted into the phenyl group instead of the cyclohexane in **ramatroban** (Figure 2).

Compound **1** showed higher selectivity for GPR44 than **ramatroban**, with a 6 times greater affinity for GPR44 than TP. However, compound **1** still displayed a weak affinity to GPR44. In an hCRTH_2_ binding assay with HEK293 cells, the K_i_ value was high at 250 nM. The compound was further found to undesirably bind to TP with a K_i_ value of 1500 nM and express a high 92% human TP inhibition at a concentration of 50,000 nM. Similar to compound **1**, a series of related compounds differing in the number and/or position of the fluorine group attached to the phenyl group was characterized (Figure 2). These compounds, however, all showed weak affinities for GPR44 with K_i_ values of 970 nM or greater, several times higher than the K_i_ value of **ramatroban** (see compounds **2**, **3**, **4**, **5**, and **6**, with K_i_ of 1300, 970, 1700, 3600, and 4200 nM, respectively). Compound **3**, which contained a 2-F phenyl group, had the lowest K_i_ value but still displayed an undesirable binding affinity (Figure 2). Overall, these compounds did not exhibit optimal properties desirable for a selective GPR44 PET tracer.

**Figure 3:**
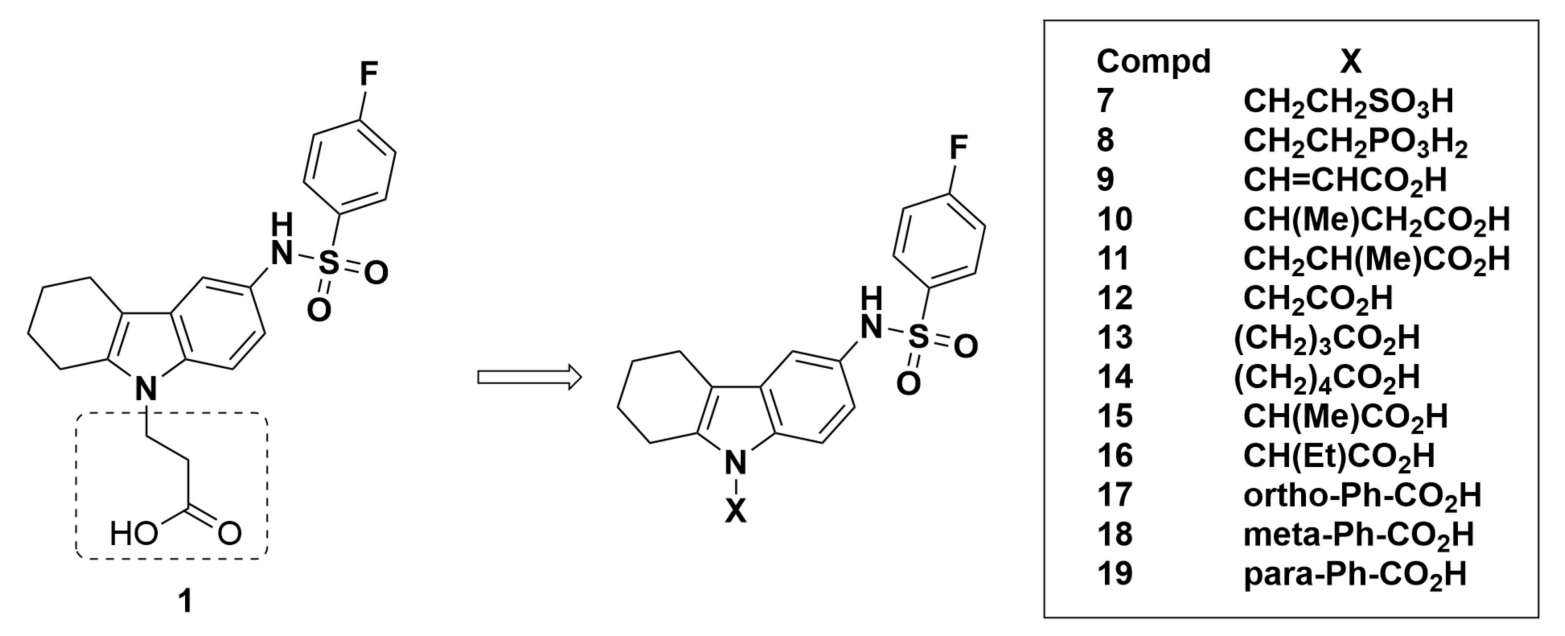
Molecular structures of compounds **7**–**19**.

Another series of compounds **7**–**11** (Figure 3) similar to compound **1** replaced the carboxylic acid group of the propanoic acid with a sulfonic acid group (compound **7**) or phosphonic acid group (compound **8**). In addition, the propanoic acid was replaced with an acrylic acid analogue (compound **9**) or with a methyl group substitution (compounds **10** and **11**). All these compounds showed a less favorable GPR44 K_i_ of 2400 nM or greater, indicating weak or no affinity to GPR44. As such, these compounds are not recommended as potential GPR44 PET probes. 

The next series of compounds explored modifications of the chain length of the propanoic acid group (Figure 3). Decreasing the chain length by one carbon to replace the propanoic acid group with an acetic acid group (compound **12**) increased the affinity for GPR44. This modification reduced the K_i_ for GPR44 to 30 nM, a significant improvement relative to **ramatroban**. Meanwhile, the affinity weakened when the chain length was increased by one carbon (compound **13**) or two carbons (compound **14**), which both resulted in poor binding to GPR44 with K_i_ values of 8300 nM and 4600 nM, respectively. Due to the better K_i_ value, compound **12** was further tested for TP selectivity and was found to demonstrate negligible off-target binding with a K_i_ > 20,000 nM and a 21% hTP inhibition at 50,000 nM compound concentration, indicating good selectivity for GPR44 over TP. Further modifications of compound **12** were made by adding either a methyl group (compound **15**) or an ethyl group (compound **16**), both of which resulted in unfavorable characteristics with K_i_ values of 4400 nM and 18,000 nM, respectively. Additional reduction in the number of carbons replaced the propanoic acid group directly with a carboxylic acid group, producing three benzoic acid isomers substituted at the ortho (compound **17**), meta (compound **18**) and para (compound **19**) position of the benzene ring (Figure 3). The compounds did not exhibit promising affinity for GPR44, with high K_i_ values of 33,000, 27,000, and 6400 nM, respectively.

**Figure 4:**
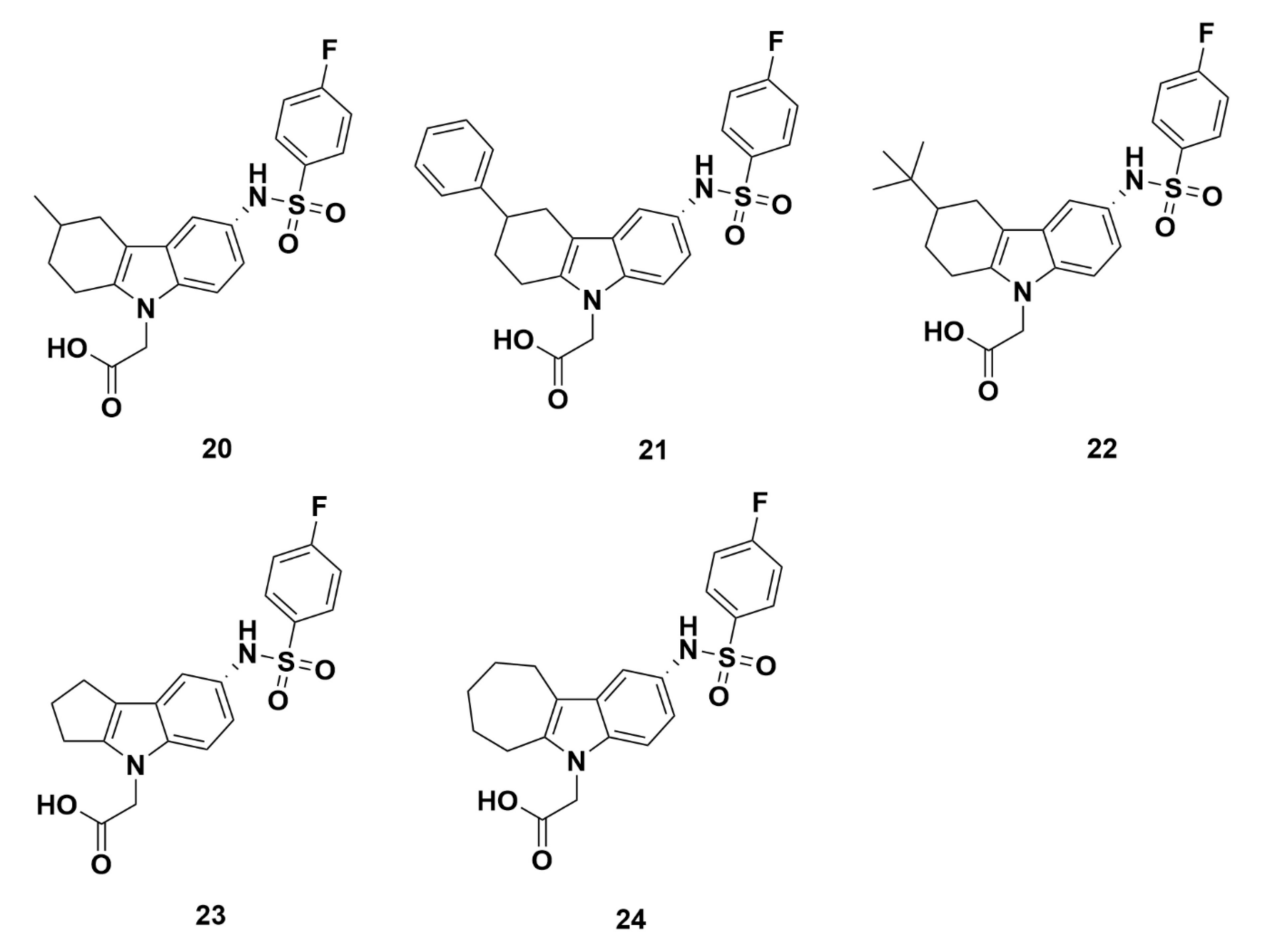
Molecular structures of compounds **20**–**24**.

The affinity and selectivity for GPR44 increased when replacing the propanoic acid group with an acetic acid group to decrease the chain length by one carbon (compound **12**, GPR44 K_i_ = 30 nM, TP K_i_ > 20,000 nM). Based on compound **12** (using the acetic acid group instead of the propanoic acid group), another group of compounds, **20**–**24** (Figure 4), had further modifications on the cyclohexane group by reducing one carbon (cyclopentane ring, compounds **23**) or adding one carbon (cycloheptane ring, compound **24**) to create a 5-membered or 7-membered ring. Compounds were also created by adding a substituent to the 6-membered ring (cyclohexane ring, compounds **20**–**22**). These compounds all showed better K_i_ values and IC_50_ values compared to **ramatroban**, in which compound **20** (addition of a methyl group to the cyclohexane ring) had the most favorable K_i_ value of 13 nM. Compound **20**’s affinity for GPR44 was further characterized in a Ca^2+^ assay measuring PGD_2_-mediated receptor activation, showing an IC_50_ of 9.7 nM. The above binding properties suggest that compound **20** binds well to GPR44. Compounds **24** (a 7-membered ring) and **21** (a phenyl group addition to the 6-membered ring) also showed favorable K_i_ values of 20 nM and 50 nM, respectively, and IC_50_ values of 22 nM and 22 nM, respectively. Compound **22** and **23**, however, are less than optimal with higher K_i_ values of 150 nM and 200 nM, respectively, and IC_50_ values of 120 nM and 130 nM, respectively. The three compounds (**20, 21,** and **24**) with the best results were further assessed for selectivity through TP binding assays, and all showed a K_i_ > 20,000 nM. Furthermore, for compounds **20** and **24**, hTP inhibition at 50,000 nM were 30% and 28%, respectively, indicating that these compounds are not likely to bind off-target to TP. However, compound **21** demonstrated an unfavorable hTP inhibition of 66%.

##### Recommendations

Based on the 2005 Robarge et al. study, compounds **12, 20, 21** and **24** are the most promising antagonists for GPR44, with strong binding affinity for GPR44 and optimal selectivity against TP. The modifications of **ramatroban** included decreasing the chain length by one carbon to replace the propanoic acid group with an acetic acid group (compound **12**), adding a methyl group to the cyclohexane group (compound **20**), adding a phenyl group to the cyclohexane group (compound **21**), and adding one carbon to the cyclohexane group to create a 7-membered ring (compound **24**). These compounds can be considered for further development as GPR44 PET tracers.

#### 2.2.2. TM-30642, TM-30643, and TM-30089

**Figure 5:**
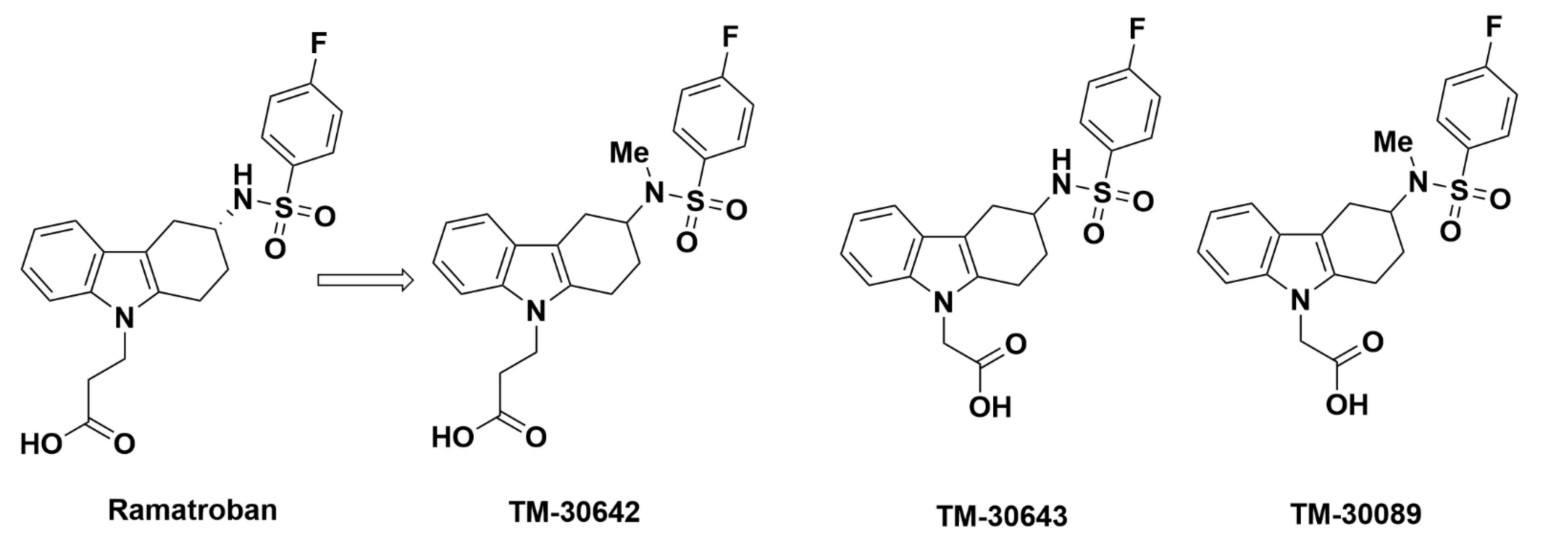
Molecular structures of TM-30642, TM-30643 and TM-30089.

Additionally, in 2005, Ulven et al. at 7TM Pharma developed a series of promising **ramatroban** analogues (Figure 5): **TM-30642**, **TM-30643**, and **TM-30089** [65].

**TM-30642** (3-{3-[(4-fluoro-benzenesulfonyl)-methyl-amino]-1,2,3,4-tetrahydro-carbazol-9-yl}-propionic acid) was synthesized by the addition of a methyl group to the nitrogen atom of the sulfonamide group in **ramatroban** (Figure 5). This structural modification was found to be advantageous, as it displayed a stronger affinity for GPR44 and weaker affinity for TP. In a [^3^H]PGD_2_ assay, **TM-30642** exhibited an optimally low K_i_ of 1.9 nM. The IC_50_ value of 26 nM, elucidated in an assay that assessed inhibition second messenger inositol phosphate or cAMP production, was adequately low. In a bioluminescence resonance energy transfer (BRET), the IC_50_ value was also low at 15 nM. **TM-30642** was determined to be a surmountable antagonist of GPR44 in [^3^H]PGD_2_ saturation isotherms, effects of which were modeled using Scatchard plots. In PGD_2_-mediated stimulation of [^35^S]GTPγS binding in transfected GPR44 cells, **TM-30642** produced rightward shifts and no change in B_max_ (maximum specific binding) in PGD_2_-stimulated inositol phosphate production, β-arrestin translocation, and ex vivo human eosinophil shape change, characteristic of competitive antagonists [55]. **TM-30642** was further tested as a racemic mixture and demonstrated negligible off-target binding for both TP and DP_1_. The weak binding to TP was found through a **[^3^H]SQ29548** assay, where **TM-30642** demonstrated a K_i_ of 3000 nM. The results were confirmed in an assessment of TP agonist U-46619-induced inositol phosphate accumulation, in which the IC_50_ was desirably high at 4600 nM, indicating that **TM-30642** had a weaker selectivity for TP than **ramatroban**. **TM-30642** was also non-selective for DP_1_, with a K_i_ of 6100 nM and IC_50_ > 10,000 nM. Of note, this study indicates that all of the enantiomers of **TM-30642** displayed negligible off-target binding to TP or DP_1_ [65]. The pharmacokinetic profile of **TM-30642** has not been made available, but a half-life of 7.72 min was reported from a [^3^H]PGD_2_ displacement binding assay.

**TM-30643** ([3-(4-fluoro-benzenesulfonylamino)-1,2,3,4-tetrahydro-carbazol-9-yl]-acetic acid) was synthesized by replacing the propanoic acid in **ramatroban** with acetic acid (Figure 5), rather than the additional methyl group in **TM-30642**. The shortening of the carboxylic acid chain in **TM-30643** led to a 1000-fold increase in GPR44 activity relative to **ramatroban** [65]. The affinity of **TM-30643** to GPR44 was high, with a K_i_ of 0.51 nM elucidated in a [^3^H]PGD_2_ assay. Along with the low K_i_ value, the IC_50_ was shown to be 3.8 nM in second messenger production and BRET assay. **TM-30643** was also characterized as an insurmountable antagonist of GPR44, in which PGD_2_ affinity does not change but B_max_ decreases upon administration. In an indirect nonequilibrium model where [^3^H]PGD_2_ association curves modeled antagonist dissociation, **TM-30643** notably reduced rate constant K_app2_ of the second slower phase of association by about 1.5 × 10^6^ times. **TM-30643** additionally did not change dissociation of [^3^H]PGD_2_ in HEK293 cells, indicating that it is unlikely to function through allosteric interactions [55]. **TM-30643** also shows a desirably weak affinity for TP, with a high K_i_ of 540 nM from a **[^3^H]SQ29548** assay. Furthermore, the IC_50_ for TP similarly showed little off-target binding at 1700 nM in an assay for U-46619-induced inositol phosphate accumulation. **TM-30643** also did not display off-target binding to DP_1_ at a K_i_ of 5300 nM and IC_50_ > 10,000 nM. Similar to **TM-30642**, **TM-30643** was studied as a racemic mixture, indicating that all enantiomers displayed little specificity to TP or DP_1_ [65]. The promising GPR44 affinity and selectivity of **TM-30643** led to further characterization of the compound. **TM-30643** exhibited concentration-dependent displacement of [^3^H]PGD_2_ in HEK293 cells, with a long half-life of 6.75 × 10^6^ min [55]. 

Encouraged by the favorable properties of **TM-30642** and **TM-30643**, Ulven and Kostenis combined the features of both analogues: addition of a methyl group to the nitrogen atom and replacement of the propanoic acid with the shorter acetic acid group. This resulted in the development of the insurmountable GPR44 antagonist **TM-30089**, also known as CAY10471 (Figure 5). Compared with **ramatroban**, **TM-30089** demonstrated stronger sub-nanomolar potency towards GPR44 and negligible off-target binding to TP and DP_1_. The affinity of **TM-30089** to GPR44 was evaluated in equilibrium competition bindings assays that showed a highly favorable K_i_ value of 0.60 nM. An IC_50_ value of 1.2 nM was also obtained through the inhibition of PGD_2_-induced cAMP production. In a GPR44 [^3^H]PGD_2_ binding assay, the log pK_i_ value of **ramatroban** was 0.58 less than the **TM-30089** value of 8.96. For TP, the **[^3^H]SQ29548** binding assay showed **TM-30089** had a log pK_i_ over 1.5 times less than that for **ramatroban**, demonstrating reduced off-target binding [66]. Similar to **TM-30642** and **TM-30643**, **TM-30089** is a racemic mixture with enantiomers that displayed significant affinity and selectivity for GPR44 over TP. **TM-30089** was found to retain its pharmacological profile in mouse and rat orthologs of GPR44. Both in vivo studies in mice and in situ studies on guinea pigs revealed that **TM-30089** inhibited characteristic responses associated with GPR44 activation. In a mouse model of allergic asthma, a 5 mg/kg oral dose of **TM-30089** applied four times over two consecutive days inhibited tissue eosinophilia and mucous cell hyperplasia, indicating strong affinity for GPR44 [66]. Furthermore, a study of circadian entrainment found that mice exhibited similarly impaired light-induced phase advance for both GPR44 knockouts and mice administered with **TM-30089** [67]. Again in a mouse model, response following oral administration of **TM-30089** mirrored GPR44 knockout mice, this time by reducing renal fibrosis [68].

##### Recommendations

Due to favorable selectivity for GPR44 over TP and DP_1_, **TM-30642**, **TM-30643**, and **TM-30089** can all be considered promising candidates for developing GPR44 PET tracers.

#### 2.2.3. Merck Analogues

##### MK-7246

**Figure 6:**
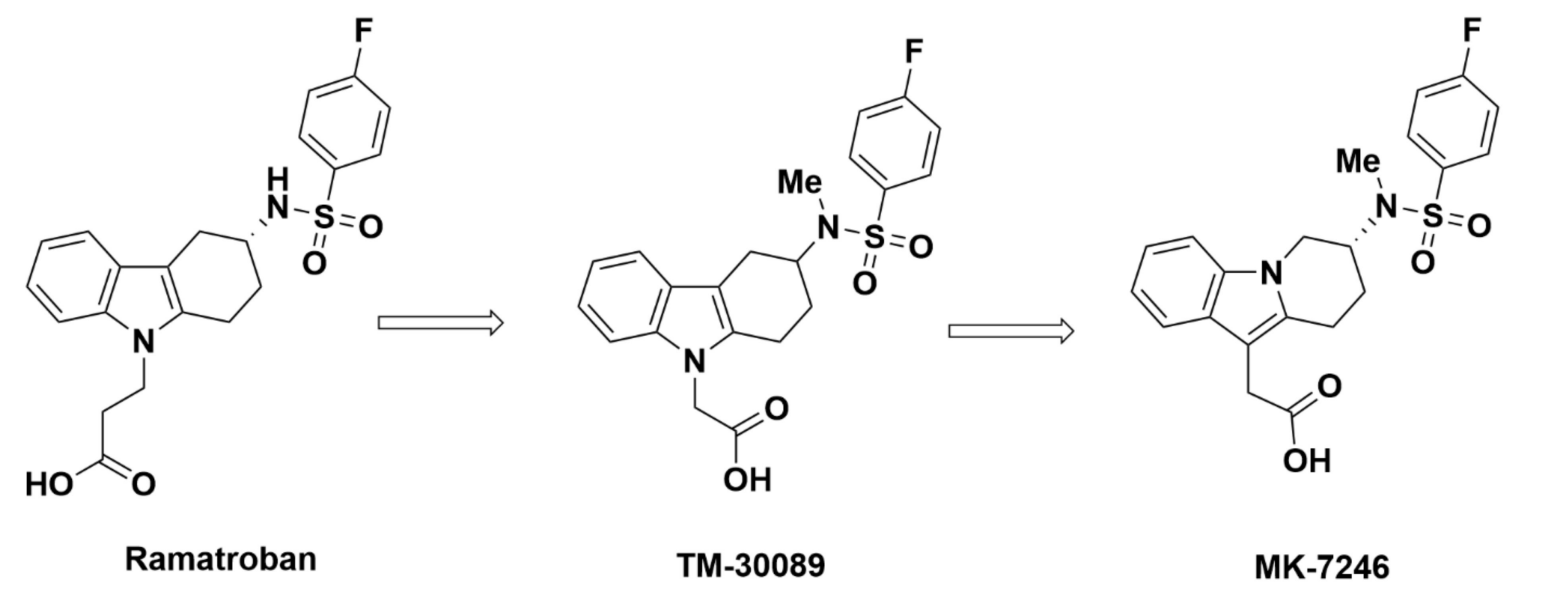
Molecular structure and synthesis of **MK-7246** from **ramatroban**.

The *R* enantiomer of the reverse indole of **TM-30089** was isolated from the racemic mixture and identified as **MK-7246** by Gallant et al. from Merck in 2007 (Figure 6), which displayed a higher affinity for and selectivity toward GPR44 over TP than the *S* enantiomer [69]. Subsequent studies investigated the compound’s pharmacokinetic and metabolic profile. **MK-7246** displayed a K_i_ value of 2.5 nM from GPR44 competition binding assays and strong binding affinity to GPR44 in recombinant mouse, rat, dog, cynomolgus monkey, and human with IC_50_ values of 9.2 nM, 4.6 nM, 6.3 nM, 6.9 nM, and 3.5 nM, respectively [70]. In addition to a favorable affinity for GPR44, **MK-7246** displayed little off-target binding, with a 149-fold weaker affinity for the DP receptor and more than 1500-fold weaker affinity for other prostanoid receptors. **MK-7246** also demonstrated antagonist potency in blocking DK-PGD_2_-induced inhibition of cAMP in recombinant cells overexpressing human GPR44 (IC_50_ = 3.0 nM), DK-PGD_2_-induced eosinophil shape change (IC_50_ = 2.2 nM), and DK-PGD_2_-induced CD11b expression on eosinophils and basophils (IC_50_ = 6.2 nM and 5.4 nM, respectively). 

Administration of **MK-7246** to mouse, rat, dog, sheep, and rhesus and cynomolgus monkey orally and intravenously demonstrated a good overall 24 h pharmacokinetic profile [70]. Favorable oral bioavailability above 50% was found in all species (F = 109%, 114%, 67%, and 57%, respectively), except in cynomolgus monkey for unknown reasons (F = 10%). While low plasma clearance following intravenous administration was observed in mouse (t_1/2_ = 2.8 h), rat (t_1/2_ = 5.6 h), rhesus monkey (t_1/2_ = 8.1 h), and sheep (t_1/2_ = 6.5 h), moderate plasma clearance was found in dog (t_1/2_ = 8.4 h). Ex vivo oral dosage of **MK-7246** revealed a relatively good relationship between **MK-7246** plasma levels and ex vivo inhibition of DK-PGD_2_-induced CD11b expression in blood eosinophils. In cynomolgus monkey, blood samples collected after oral administration of **MK-7246** showed an IC_50_ value of 15 nM, and direct addition of **MK-7246** to blood in vitro showed an IC_50_ value of 3.5 nM. In sheep, the IC_50_ values obtained were 107 nM following intravenous administration and 22.5 nM after direct in vitro administration. Overall, these results demonstrate that **MK-7246** exhibits strong affinity and selectivity for human GPR44, antagonist activity on recombinant and endogenous GPR44, and good oral bioavailability and metabolic stability in various species.

Additional studies on the association kinetics of **[^3^H]MK-7246** from recombinant human GPR44 measured an on rate of association (K_on_) value of 0.0016 to 0.0017 min^−1^ × nM^−1^ and a t_1/2[on]_ value of 19.8 to 20.9 min [70]. Comparatively, dissociation of **[^3^H]MK-7246** had an off rate of dissociation (K_off_) value of 0.0212 to 0.0216 min^−1^ × nM^−1^ and the t_1/2[off]_ value of 32.2 to 33.9 min. Given the slower dissociation than association, these studies derived a low K_d_ (K_off_/K_on_) of 13.6 to 18.6 nM for **[^3^H]MK-7246** at GPR44. In contrast, saturation analysis revealed a K_d_ value of 2.3 nM for **[^3^H]MK-7246**, which is similar to the **MK-7246** K_i_ value of 2.5 nM from competition binding assays.

In vitro and in vivo PET studies performed for **[^11^C]MK-7246** also demonstrated favorable results [71]. Autoradiography assays on human pancreatic sections with and without T2D showed receptor-mediated focal binding in areas with insulin positive islet of Langerhans, indicating the potential of **[^11^C]MK-7246** to accurately quantify BCM. In rat, **[^11^C]MK-7246** demonstrated rapid distribution in the blood, rapid excretion from the liver, and more gradual excretion through the kidney following intravenous administration. Most other tissues demonstrated low uptake and rapid washout. Biodistribution assays in rat by PET-MRI imaging predicted through dosimetry calculations a low radiation dosage in human, especially in radio-sensitive tissues such as red marrow. The largest predicted absorbed radiation doses were in the myocardium, liver, and small intestine of humans. Overall, the absorbed dose for the whole body was approximated as 0.0036 mSv of **[^11^C]MK-7246**. This could potentially allow for up to 7 PET examinations per year before reaching the clinical research annual limit of 10 mSv. In pig studies, **[^11^C]MK-7246** showed similar excretion behavior from the liver and kidney and strong uptake from the small intestine. PET-CT investigations in pig demonstrated GPR44-mediated binding to the pancreas and off-target binding to the spleen. However, weaker binding was observed in the human spleen indicating that off-target spleen binding is not expected for human PET imaging. Abolishment of baseline **[^11^C]MK-7246** uptake in pig using **MK-7246** pretreatment showed a 66% reduction in the pancreas and 88% reduction in the spleen. While strong **[^11^C]MK-7246** concentrations in the liver, duodenum, and jejunum may spillover into the PET imaging of small animals, the issue was not observed in pig PET imaging, suggesting that it may not pose a problem in human imaging. 

Excretion of **[^11^C]MK-7246** was found to occur mainly through the liver, bile, and intestines in both rat and pig studies [71]. The major metabolite excreted in a rat study was acyl glucuronide (90%), which displayed weak affinity for GPR44 (K_i_ > 7200 nM). Additionally, excreted were minor amounts of the parent **MK-7246** (2.7%) and a decarboxylated metabolite (2.3%) [69]. Acyl glucuronide demonstrated low to moderate plasma clearance of 2.2–15 mL/min/kg, normal volume distribution of 0.5–4.8 L/kg, and normal plasma half-life from 5.6 to 11 h. The plasma exposure of acyl glucuronide compared to **MK-7246** was found to vary among species and be highest in human primate (0.5–1.5) in comparison to dog (0.06) and Sprague Dawley rat (0.02). In various animals, the order of metabolic stability has been characterized as decreasing from the Sprague Dawley rat, beagle dog, cynomolgus monkey, to rhesus monkey and human. 

##### Recommendations

In addition to having been well characterized, **MK-7246** advantageously demonstrates potency and selectivity for GPR44 over other prostanoid receptors.

##### Merck Analogues in 2008

**Figure 7:**
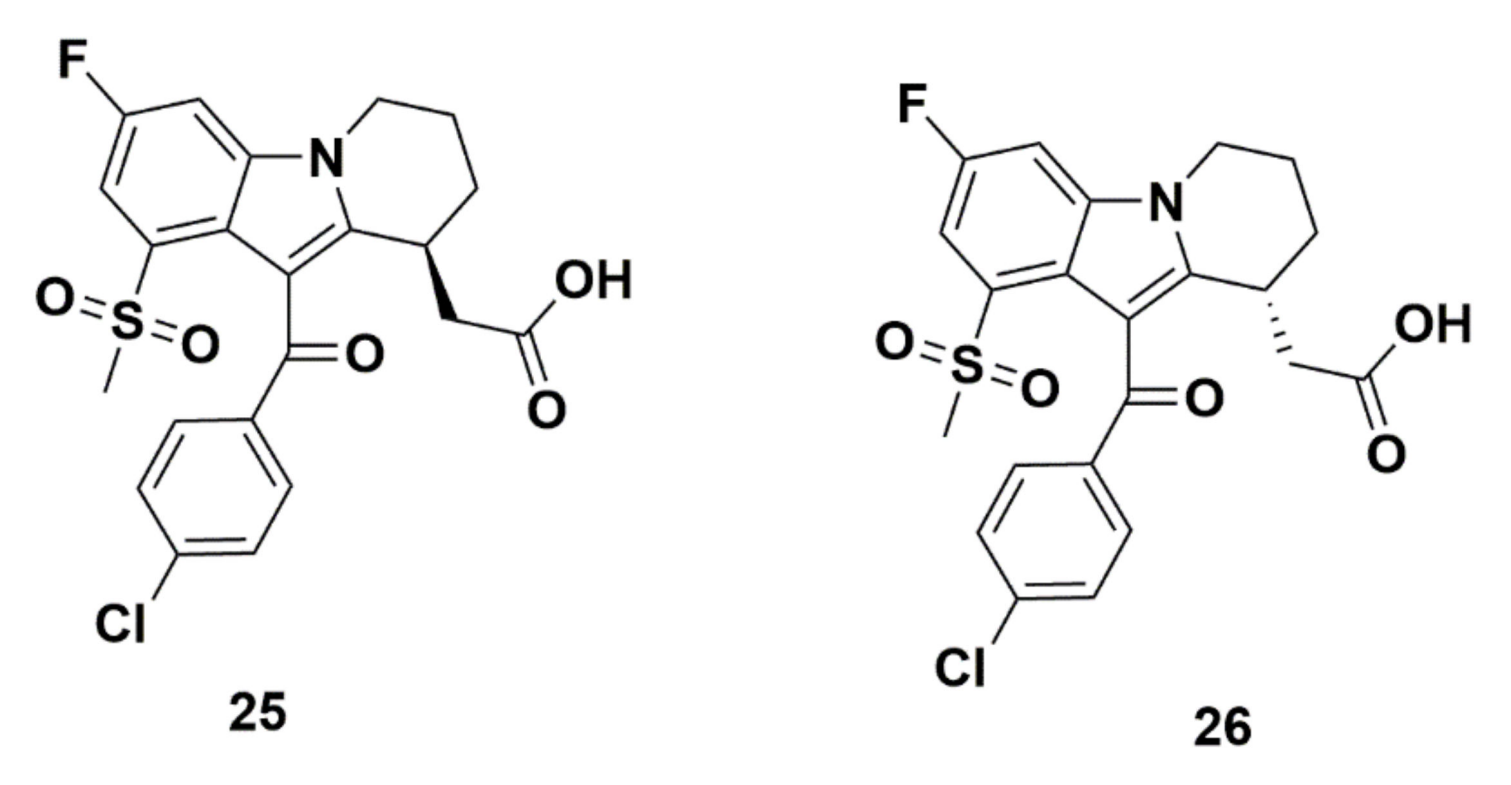
Molecular structures of compounds **25** and **26**.

In 2008, a series of DP_1_ antagonists, described by Beaulieu et al. at Merck, were developed based off a 6-5-5 or 6-5-6 tricyclic scaffold. Among those, two enantiomers with a tetrahydrocarbazole group were synthesized (compounds **25** and **26**, Figure 7), but the compounds exhibited poor affinity for GPR44. This may be inferred by the design of the study to develop DP_1_ antagonists. The binding for one could not be elucidated (compound **25**), whereas the other demonstrated binding at 19,200 nM (compound **26**). On the other hand, both exhibited strong affinity to DP_1_ [72]. Due to weak binding to GPR44 and off-target binding to DP_1_, these compounds should not be considered for development of GPR44 PET tracers. Other compounds in this series do not contain a tetrahydrocarbazole scaffold and, therefore, are not discussed in this study.

##### Merck Analogues in 2011a

Encouraged by the promising results from **MK-7246**, Zaghdane et al. at Merck synthesized a series of novel GPR44 antagonists in 2011. These compounds were based on the structure of **MK-7246**, except with an amide group replacing the sulfonamide group [73].

**Figure 8:**
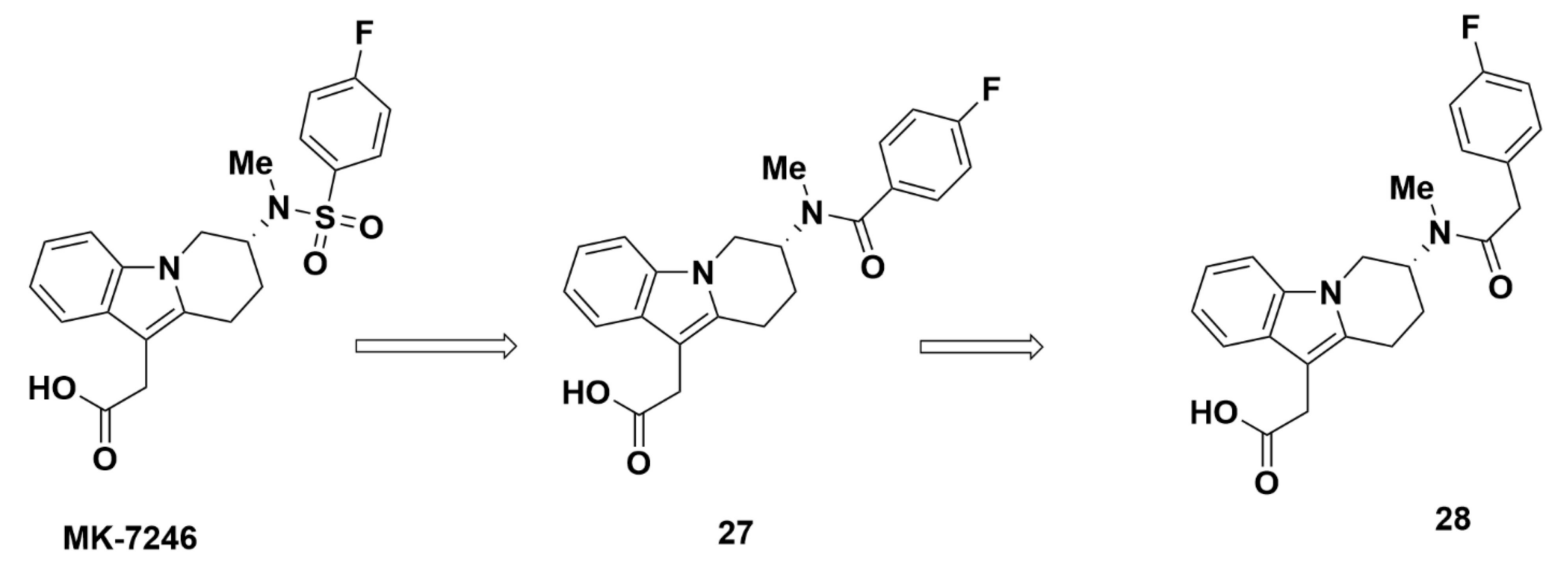
Molecular structures of compounds **27** and **28**.

The replacement of the sulfonyl group of **MK-7246** with a carbonyl group yielded compound **27**. Further addition of a CH_2_ group between the carbonyl and phenyl group produced compound **28**, which showed promising properties (Table 1). The binding affinity of compound **28** for GPR44 was desirably strong with a K_i_ of 7.4 nM and IC_50_ of 12 nM, characterized through a cAMP assay in HEK293 cells. Additional studies for GPR44-related eosinophil shape change confirmed strong affinity with an IC_50_ at 4.8 nM. The selectivity of the compound was found to be optimal with IC_50_ values for DP_1_ and TP above 1000 nM. The pharmacokinetic profile of compound **16** showed issues with bioavailability, however, possibly due to limited access into the liver. In rat, the bioavailability was only 14%, but the clearance, distribution, and half-life were comparable to **MK-7246** (0.38 mL/min/kg, 0.13 L/kg, and 4.6 h, respectively). The compound was also predicted to have no issues with drug-drug interaction, as there was little selectivity for CYP (IC_50_ > 50,000 nM). The metabolism of compound **28** was low, with 91% concentration remaining after 1 h and 60% glucuronidated. The study identified no hydroxylated metabolites produced from the compound or amide hydrolysis. However, compound **27** showed a weak affinity for GPR44 (Figure 8). The K_i_ and IC_50_ elucidated through a cAMP assay both were unsuitably high at 340 nM and 2500 nM, respectively. As such, the poor affinity of this compound led to discontinuation of its testing.

**Figure 9:**
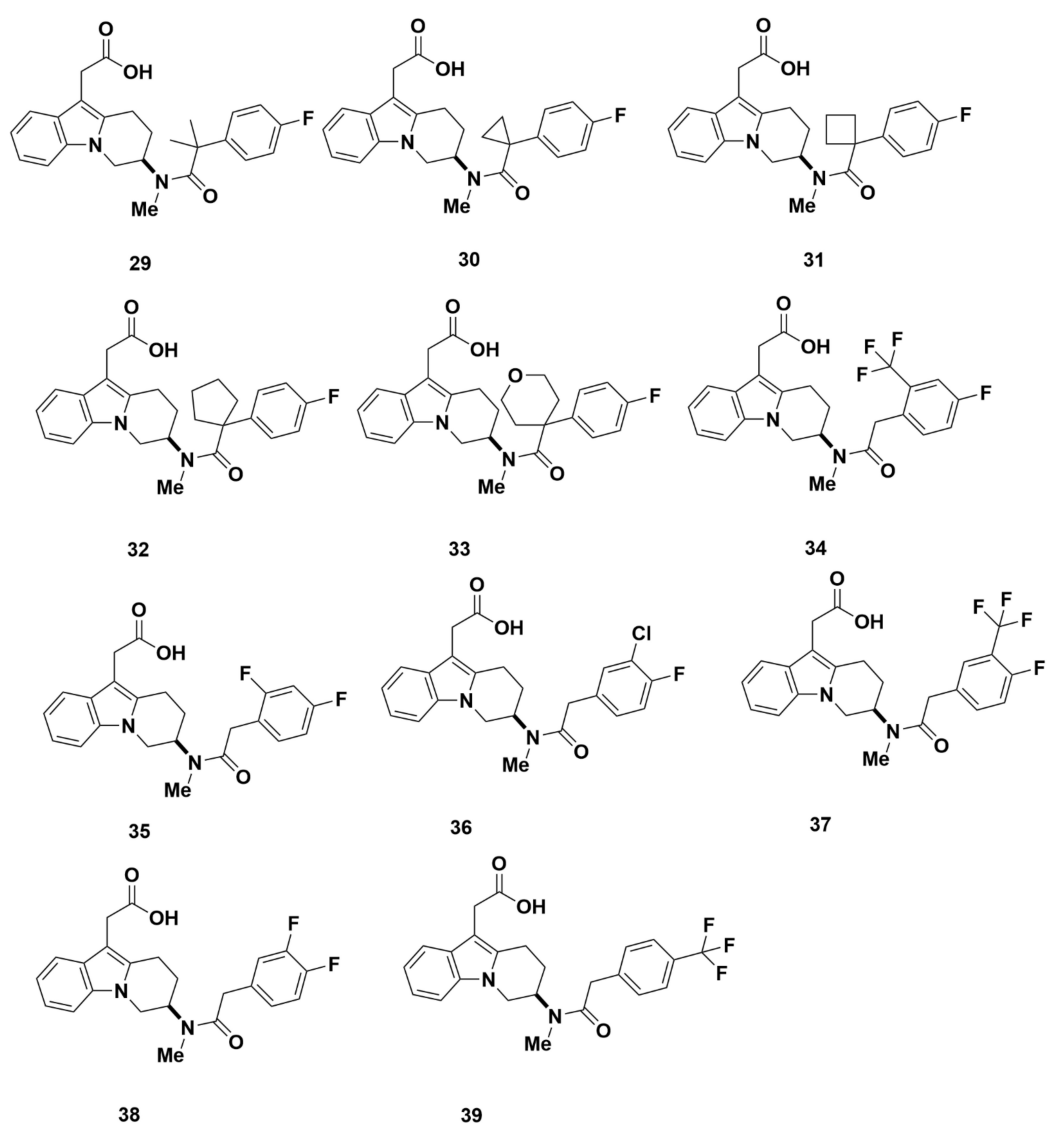
Molecular structures of compounds **29**–**39**.

Methylation and replacement of the sulfone group by two methyl groups (**29**), a C3 (**30**), C4 (**31**) or C5 (**32**) cyclic group, or an oxygen containing 6-membered ring (**33**) produced compounds with optimal GPR44 affinity (Figure 9). The K_i_ values were favorably low at 3.0 nM, 2.0 nM, 1.8 nM, 1.7 nM, and 4.3 nM, respectively, with a comparable eosinophil shape change IC_50_ values of 6.0 nM, 4.4 nM, 1.0 nM, 1.4 nM and 2.4 nM. Furthermore, cAMP IC_50_ values of 6.2 nM, 2.0 nM, 4.0 nM, 4.0 nM and 8.0 nM for compounds **29**, **30**, **31**, **32** and **33**, respectively, confirmed that these compounds have a strong affinity for GPR44. Additional characterization reported that compounds **31**, **32**, and **40** (discussed below) have superior pharmacokinetic properties, especially oral bioavailability. For instance, compound **31** showed a high bioavailability (F = 93%). Further properties (Cl = 12 mL/min/kg, V_d_ = 2.6 L/kg, t_1/2_ = 2.6 h, and CYP3A4 and CYP C9 IC_50_ > 50,000 nM) were also in the optimal range.

Additional compounds were synthesized by substitution of the phenyl ring outside the tetrahydrocarbazole group but were not further characterized because they exhibited weak affinity for GPR44 (Figure 9). The K_i_ values were 141 nM (compound **34**), 70 nM (compound **35**), 22 nM (compound **36**), 126 nM (compound **37**), 19 nM (compound **38**) and 408 nM (compound **39**).

**Figure 10:**
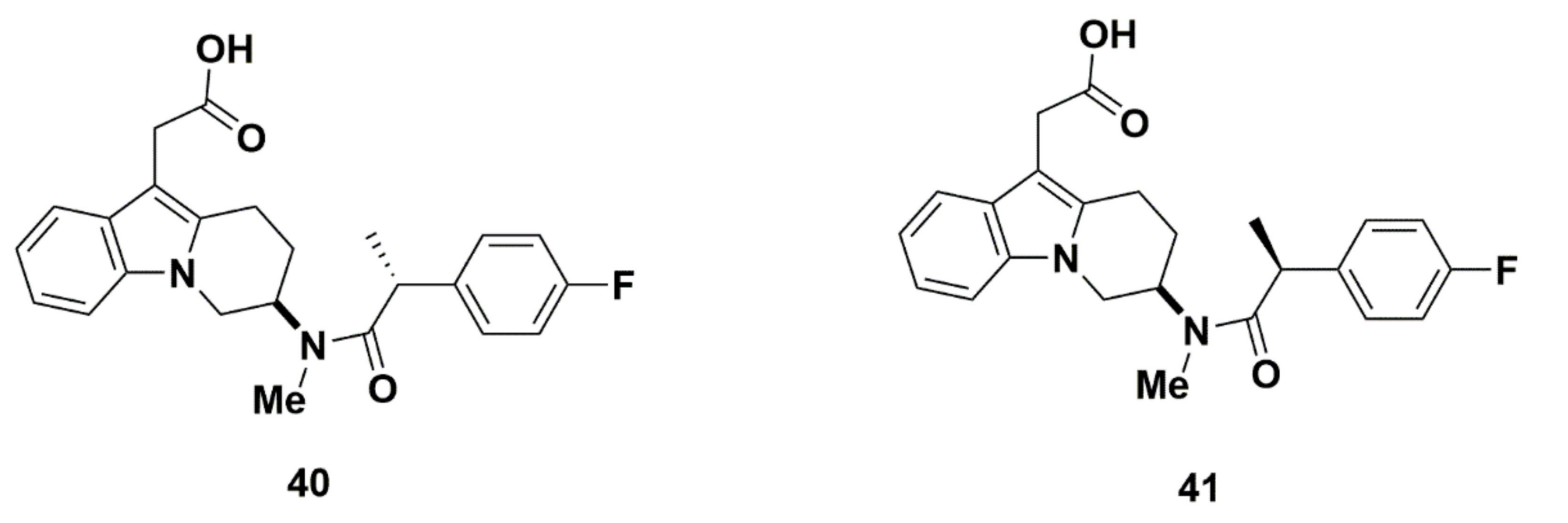
Molecular structures of compounds **40** and **41**.

Addition of a methyl group resulted in two diastereomers, but only the *R*-isomer (compound **40**) exhibited favorable affinity values (Figure 10). The K_i_ value was low at 4.0 nM, and the IC_50_ values, elucidated through eosinophil shape change and cAMP production, were 0.96 nM and 3.0 nM, respectively. The selectivity of compound **40** for GPR44 was also promising, in which the compound exhibited at least 500-fold selectivity for GPR44 over IP, EP_1_, EP_2_, EP_3_, EP_4_ and FP. In vivo studies on compound **40** found optimal pharmacokinetic properties. The bioavailability (F) of 73%, drug clearance (Cl) of 1.1 mL/min/kg, volume of distribution (V_d_) of 0.36 L/kg, and half-life of 4.7 h were all optimal or acceptable. Compound **40** should also have little drug-drug interaction, as its affinity for CYP 3A4 and CYP 2C9 showed an IC_50_ greater than 50,000 nM. By comparison, the other isomer (compound **41**) showed a higher K_i_ value of 39 nM and IC_50_ value of 30 nM from a cAMP assay.

##### Recommendations

The strong affinity and pharmacokinetic properties mark compounds **29**–**33** and **40** as promising candidates for PET tracers, with compounds **31** and **32** showing the most favorable K_i_ values. Therefore, all these compounds may be developed into potentially selective GPR44 PET tracers.

##### Merck Analogues in 2011b

In 2011, Simard et al. at Merck synthesized a series of compounds based on **MK-7246** [74].

Compounds **42**–**45**, which are regioisomers of one another with different placements of the N atom in the pyridine ring, were found to have weak affinities for GPR44 (Table 2). The affinity was most promising for compound **45**, which has a 7-azaindole group, with a K_i_ value of 3.3 nM in binding assays. However, the K_i_ values were unfavorable for the other three compounds at 7189 nM (compound **42**), 6725 nM (compound **43**), and 139 nM (compound **44**). The cAMP IC_50_ values correlated to the K_i_ values and were undefined (compounds **42** and **43**), 351 nM (compound **44**), and 3.4 nM (compound **45**). Further characterization of compound **45** through the eosinophil shape change assay reported an IC_50_ value of 7.0 nM. There was also little to no off-target binding to TP and DP_1_. Specifically, the K_i_ of compounds **42**–**45** to TP were > 7000 nM, > 6800 nM, > 22,000 nM, and > 22,000 nM, respectively; the K_i_ for binding to DP_1_ were > 1300 nM, > 3400 nM, > 12,000 nM, and > 47,000 nM, respectively. Due to its strong affinity and selectivity for GPR44, compound **45** was further characterized for covalent binding, and the resulting value (52 pmol equiv/mg at 6 h and 50 equiv/mg at 24 h) indicated potential hepatotoxicity. Meanwhile, the CYP value was optimal at an IC_50_ > 50,000 nM, indicating little potential for drug-drug interaction. 

**Figure 11:**
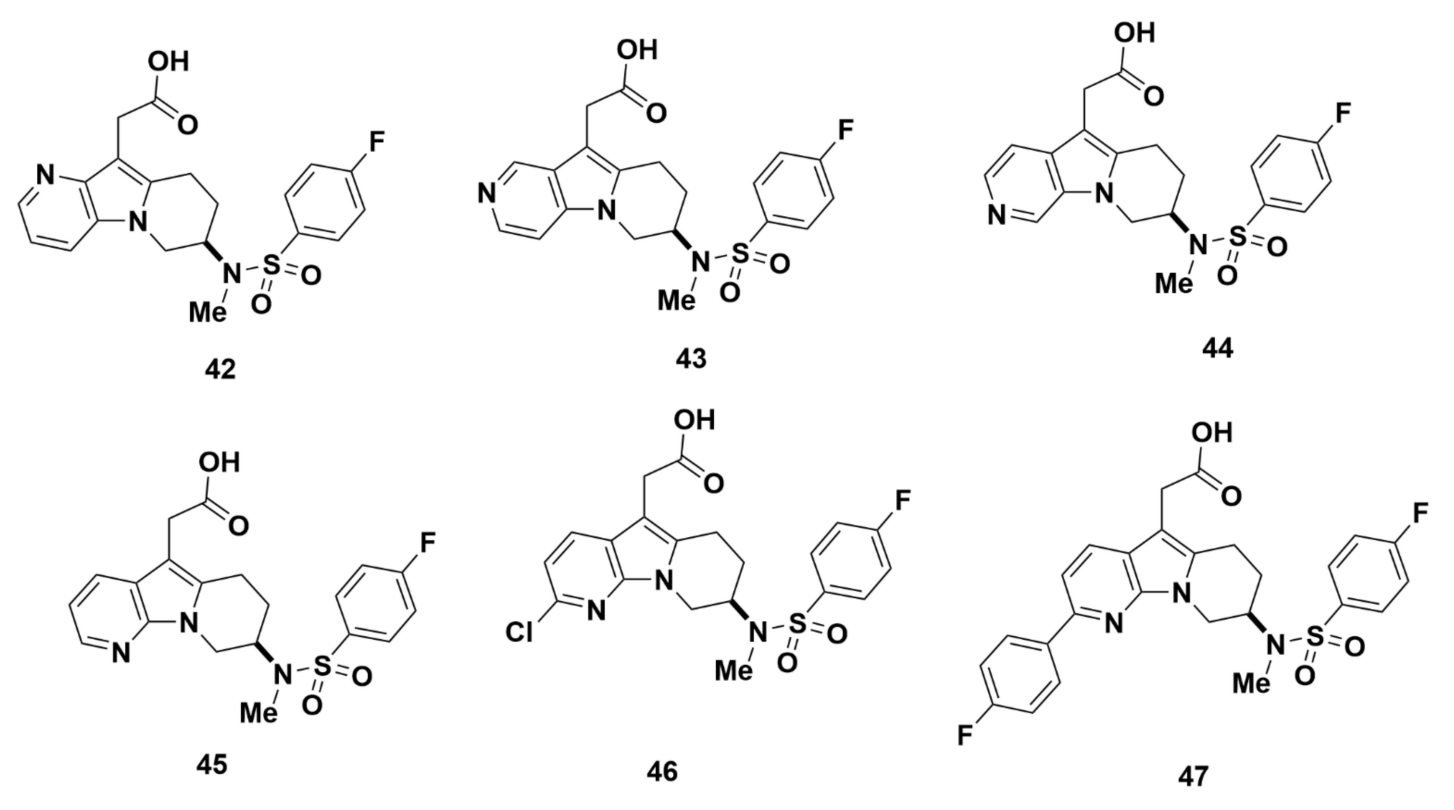
Molecular structures of compounds **42**–**47**.

Compound **46** is a **ramatroban** analogue with a chlorine group that creates an electrophilic site on the azaindole. It exhibited strong affinity for GPR44 with a K_i_ value of 1.8 nM. Another compound with a fluorine substituted phenyl group in a corresponding position to the chlorine of compound **46** (compound **47**) showed similarly strong affinity of K_i_ = 1.9 nM. The IC_50_ values, determined in a cAMP assay, were similar at 3.2 nM (compound **46**) and 3.6 nM (compound **47**). However, in eosinophil shape change assays, compound **46** exhibited a more favorable IC_50_ of 3.3 nM than the IC_50_ of 15.8 nM of compound **47** (Figure 11). All K_i_ values for TP and DP_1_ were greater than 1000 nM (compound **46**: TP > 27,000 nM and DP > 18,000 nM; compound **47**: TP > 1000 nM and DP > 3800 nM). Both compounds demonstrated favorable affinity and specificity for GPR44, with negligible binding to TP and DP_1_.

**Figure 12:**
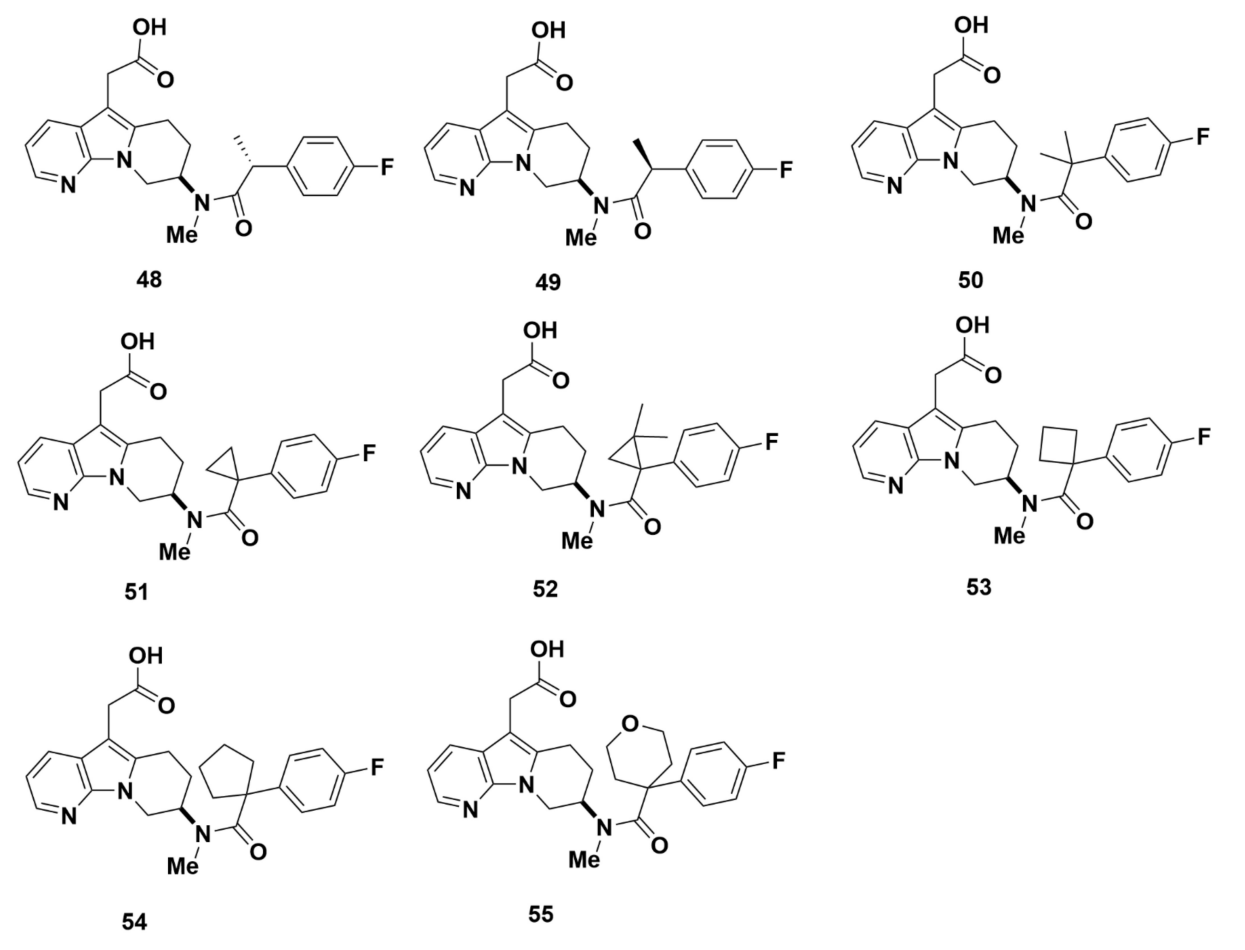
Molecular structures of compounds **48**–**55**.

Compounds **48**–**55** (Figure 12) were produced with an amide group and displayed optimal binding affinity. All K_i_ values were 11.5 nM or lower, except for compound **49**, with the lowest value being 3.4 nM for compounds **48** and **53** (Table 2). The strong affinity indicated by the low K_i_ values was corroborated further in cAMP and eosinophil shape change assays, with IC_50_ values under 10 nM. All compounds showed little potential for off-target binding, as K_i_ values for DP_1_ and TP were all greater than 4000 nM. Unlike compound **48**, however, the stereoisomer compound **49** had cAMP assay K_i_ and IC_50_ values of 21.1 nM and 73.8 nM, respectively, indicating weak affinity for GPR44. The difference between compounds **48** and **49** evidenced the stereo-sensitivity of GPR44 binding. 

The pharmacokinetic properties of the compounds were assessed in rat and demonstrated greater than 50% bioavailability in only three compounds. Compounds **48**, **50** and **51** exhibited bioavailability of 84%, 156% and 63%, respectively. The pharmacokinetic profile of compound **48** was the foremost with a rapid clearance of 3.6 mL/min/kg, a low volume of distribution of 0.67 L/kg, and a half-life at 4 h. Based on its favorable pharmacokinetic profile, compound **48** was further tested for covalent binding (8 pmol equiv/mg at 6 h and 6 pmol equiv/mg at 24 h), CYP binding (IC_50_ > 50,000 nM), and K_obs_ (activation constant, representing CYP3A4 time-dependent inhibition, of < 0.004 min^−1^ at 50 mM). The lowered K_obs_ implies that compound **48** may have less risk for irreversible binding to the cytochrome active site and subsequent drug-drug interactions. 

##### Recommendations

Compounds **45**–**48** and **50**–**55**, particularly compounds **48** and **53**, demonstrated desirable affinity and selectivity for GPR44. These compounds may be considered as candidates for developing GPR44 PET tracers to assess BCM.

#### 2.2.4. AstraZeneca

**Figure 13:**
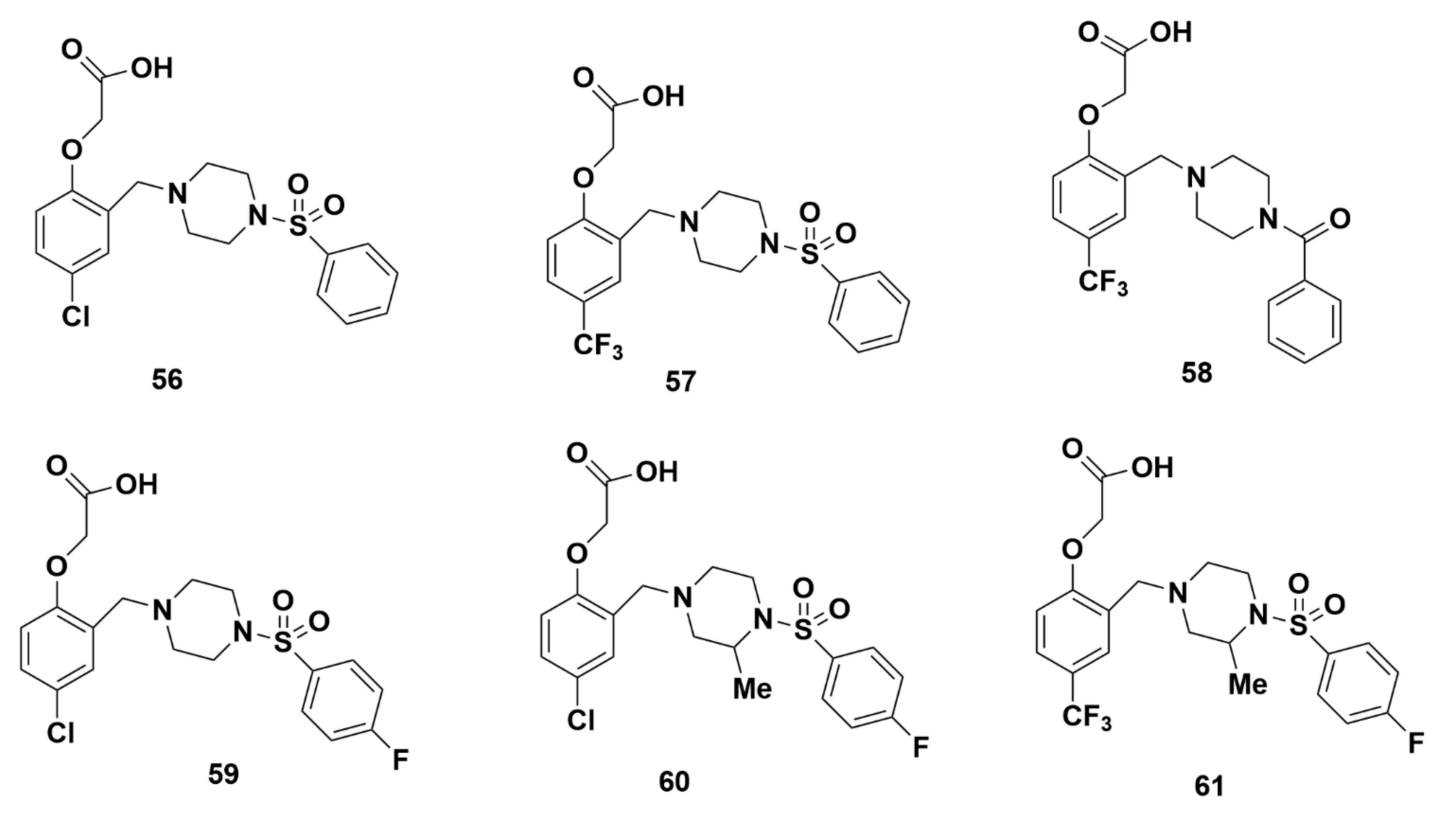
Molecular structures of compounds **56**–**61**.

In 2011, Luker et al. at AstraZeneca conducted a virtual screening to identify compounds with similar pharmacophore features as **ramatroban** [75]. The identified lead compound **56** (Figure 13) was then optimized through side chain modifications and changes around the acid to develop a series of zwitterions which included potent and selective GPR44 antagonists. Although compound **56** shows a moderate IC_50_ for GPR44 of 16 nM, compound **56** is not discussed in more detail as it does not contain any fluorine atoms for potential ^18^F-labeling.

Compound **57**, which replaced the -Cl of compound **56** with a para-CF_3_ subunit on the phenoxyacetic acid ring, had a favorable IC_50_ for GPR44 of 18 nM. It was further confirmed as an antagonist, rather than an agonist, of GPR44 through an eosinophil shape change assay, where it lowered the response rate of DK-PGD_2_ to less than 10%. The lipophilicity, measured through log D_7.4_ of 0.4, and clearance (both in rat hepatocytes and human liver microsomes) of < 4 µL/min/1 × 10^6^ cells and 6 µL/min/mg, respectively, were both moderate. However, in a Ca^2+^ assay, the IC_50_ for compound **57** was found to be unacceptably high at 517 nM.

Compound **58** was produced through replacement of the sulfonyl group in compound **56** with a carbonyl group, and replacement of the chlorine group with the CF_3_ group [75]. Importantly, this resulted in minimal changes to potency, despite the expectation that the highly charged acids would require control of polar surface area in order to maintain optimal oral pharmacokinetic profiles [76]. Compound **58** demonstrated an IC_50_ value of 316 nM in radiometric binding assays [75]. As such, compound **58** showed an undesirably weak affinity for GPR44 (Table 3).

The halogen substitution on the distal aryl ring using 4-fluorophenyl resulted in compound **59** (Figure 13), which demonstrated a low IC_50_ value of 10 nM. An additional substitution with a methyl group on the piperazine ring (compound **60**) further decreased the IC_50_ to a desirably low value of 1.0 nM (Figure 13). The para-position was found to be more potent and metabolically stable. Replacement of the chlorine in compound **60** with a CF_3_ (compound **61**) maintained similar activity, with an IC_50_ value of 0.3 nM (Figure 13). While other compounds did not display agonist activity and behaved as antagonists, compound **61** showed partial agonist activity in the eosinophil shape change assay, with a DK-PGD_2_ efficacy of around 40%.

##### Recommendations

The strong affinities of compounds **59**–**61** make them promising candidates for the development of GPR44 PET tracers.

#### 2.2.5. Actelion Pharmaceuticals Ltd.

##### Setipiprant

In 2013, Fretz et al. at Actelion Pharmaceuticals Ltd. used a lead optimization program to screen 80,000 compounds from a GPCR library, looking for potential GPR44 antagonists. In screening for structure–activity relationship (SAR), particularly for oral bioavailability, the study discovered the precursor to a lipophilic naphthyl-containing compound, later known as **setipiprant**. **Setipiprant** (2-(2-(1-Naphthoyl)-8-fluoro-3,4-dihydro-1*H*-pyrido[4,3-b]indol-5(2*H*)-yl)acetic acid or ACT-129968) is a derivative of indole-1-acetic acid, which is a selective inhibitor of GPR44 [77].

The GPR44 binding affinity of **setipiprant** was found to be promising. In HEK-293 cells expressing hCRTH_2_, Ca^2+^ and cAMP assays showed IC_50_ values of 30 nM and 80 nM, respectively. Additional binding studies in buffer (6 nM) and HSA (340 nM) for IC_50_ were also characterized, confirming desirable affinity. The selectivity of **setipiprant** to GPR44 was also encouraging, as IC_50_ values for other prostanoid receptors were consistently above 1200 nM. Specifically, a screen for DP_1_ (IC_50_ = 1290 nM, f_Sel_ = IC_50_ (hDP_1_)/IC_50_ (hCRTH_2_) = 215), EP_1_ (IC_50_ > 25,000 nM), EP_2_ (IC_50_ = 2600 nM), EP_3_ (IC_50_ > 25,000 nM), EP_4_ (IC_50_ > 10,000 nM), and TP (IC_50_ > 25,000 nM) binding showed weak affinity for prostaglandin receptors beside GPR44. **Setipiprant** is therefore a selective and potent inhibitor of GPR44.

The physicochemical properties of **setipiprant** were also identified, revealing a molecular weight of 402.42 Da. The elimination half-life of **setipiprant** was shown to be greater than 4 h following incubation in rat and human plasma and simulated intestinal fluid (SIF), but only greater than 1 h in simulated gastric fluid (SGF). **Setipiprant**’s intrinsic clearance was favorable at Cl values below 10 uL/min^/^mg/protein. Additional properties of low lipophilicity and high solubility were also regarded as optimal, at a ligand efficiency (LE) of 0.37, cLogP of 3.3, log D_7.4_ of 0.1, solubility in water of 50 ug/mL at pH 4.3, solubility in buffer of 90 ug/mL at pH 4, and solubility in buffer of 880 ug/mL at pH 7. An in vivo study using rats found intravenously administered **setipiprant** AUC_(0−last)_ of 58,500 ng/h/m, plasma clearance of 1.3 mL/min/kg, and oral bioavailability of 44%. Later, in male beagle dog administered **setipiprant** orally and intravenously, the compound showed an AUC_(0−last)_ of 91,100 ng/h/m, clearance of 1.3 mL/min/kg, and bioavailability of 55%. The higher exposure and slightly greater bioavailability but same clearance in dog and rat shows that **setipiprant** exhibits species-dependent pharmacokinetic properties. Of note is that a FLIPR assay against mouse, rat, guinea pig, and dog GPR44 revealed that **setipiprant** acts as a partial agonist on mouse and guinea pig receptors. This indicates that studies in vivo may be limited in choice of organisms.

The possibility of drug-drug interaction was shown to be low, with an in vitro CYP2C9 IC_50_ > 50,000 nM, HLM of 5 μL/min/mg protein, RLM of 5 μL/min/mg protein, and intrinsic clearance (Cl_int_) in rat hepatocytes of 3.1 μL/min/10^6^ cells. **Setipiprant** was also found to not accumulate in the body, as assessed by clinical trial [78]. The profile of **setipiprant** was further characterized in an open-label, 2-period, 2-way crossover, randomized study in which patients were administered 250 or 500 mg **setipiprant**, and elucidated the following properties in humans (Table 4) [79].

As a result of its promising properties, **setipiprant** continued to be assessed in clinical studies. A 3-centre, double-blinded, placebo-controlled, cross-over study of 18 allergic asthmatic males administered with 1000 mg **setipiprant** or matching placebo showed that in allergen-induced airway responses, the compound reduced the late asthmatic response, inhibiting the AUC on average by 25.6%, while no difference in the early asthmatic response or allergen-induced changes in exhaled nitric oxide was observed. **Setipiprant** also showed a protective effect against the allergen-induced airway hyperresponsiveness to methacholine [80]. A prospective, randomized, double-blind, placebo- and active-referenced (cetirizine) phase 2 trial and phase 3 trial of 100–1000 mg **setipiprant** investigated effects on allergic rhinitis, and showed significant, dose-related improvement in mean change from baseline day-time nasal symptom scores over 2 weeks for the phase 2 trial but no significant effect in the phase 3 trial. Additionally, total and individual night-time nasal symptom scores and day-time eye symptom scores symptom scores were significantly improved with **setipiprant** 1000 mg compared to placebo in the phase 2, but not the phase 3, trial. The study did show a favorable safety profile for **setipiprant** [81]. Another randomized, double-blind, placebo-controlled study of 2000 mg **setipiprant** administered orally also exhibited favorable safety in both single- and multiple-dose administration [78].

The inadequate efficacy exhibited by **setipiprant** as a pharmaceutical in the described phase III study, in addition to three phase II studies (including a 12-week study in 438 asthmatics), led to the termination of further studies in April 2012 [82].

**Figure 14:**
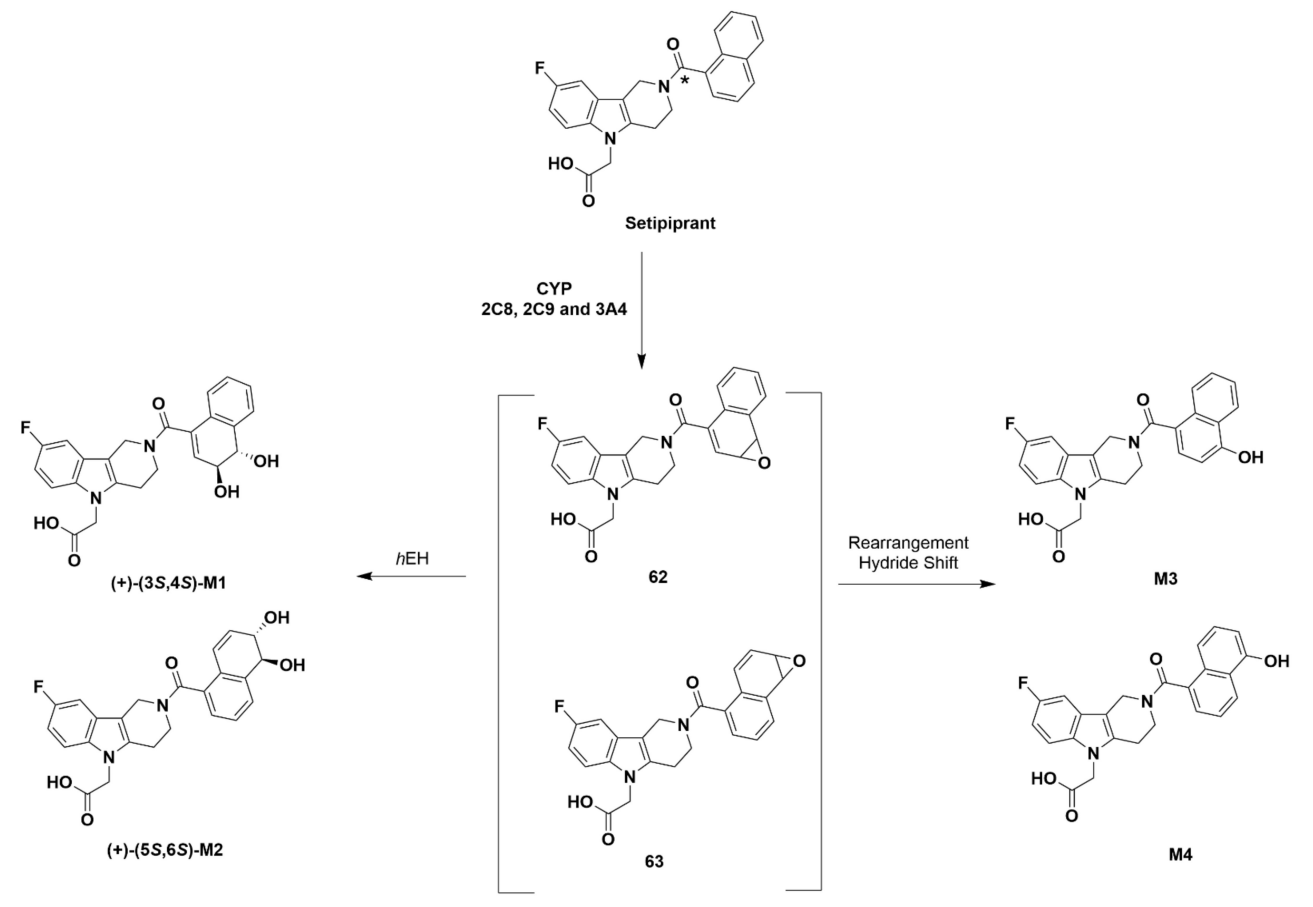
Metabolites of **setipiprant** [83]. The asterisk (*) denotes the position of the ^14^C atom in **[^14^C]setipiprant**.

Later studies characterized the metabolic breakdown of **setipiprant**, which discovered four main metabolites (**M1-4**, Figure 14). The metabolites were found following incubation of **[^14^C] setipiprant** with human hepatocytes, microsomes, and bactosomes expressing the human enzymes CYP2C8, CYP2C9, and CYP3A4. The CYP enzymes initially epoxide the naphthoyl ring of **setipiprant**, and the regioisomeric epoxide products are hydrated by human epoxide hydrolase **62** to produce **M1** and changed by epoxide **63** to produce **M2**. The pre-hydration epoxide may also be subject to a hydride shift (NIH shift) that produces 4-hydroxy-naphthoyl metabolite **M3**. Other processes produce the 5-hydroxy-naphthoyl metabolite **M4** [83].

A clinical study of 6 healthy male subjects orally dosed with a total of 1000 mg ^14^C-labeled **setipiprant** also showed that **M1** and **M2** were the main metabolites. **M1** and **M2** accounted for 20.0% and 15.3% of the administered radioactive dose. Of note is that **M2** and other metabolites besides **M1** were not determined in plasma, and even **M1** exhibited concentrations consistently below 10% of those of unchanged **setipiprant**. Feces proved to be the primary recovery route for **setipiprant**, although the recovered amount of unchanged **setipiprant** in urine did account for 3.7% [84]. A second clinical study generally assessed that **setipiprant** was eliminated through a biphasic pattern with an elimination half-life between 10 h and 18 h, where steady-state conditions were reached after 2–3 days [78]. Due to the long elimination half-life, the metabolism of **setipiprant** is not expected to be a concern for PET imaging.

**Figure 15:**
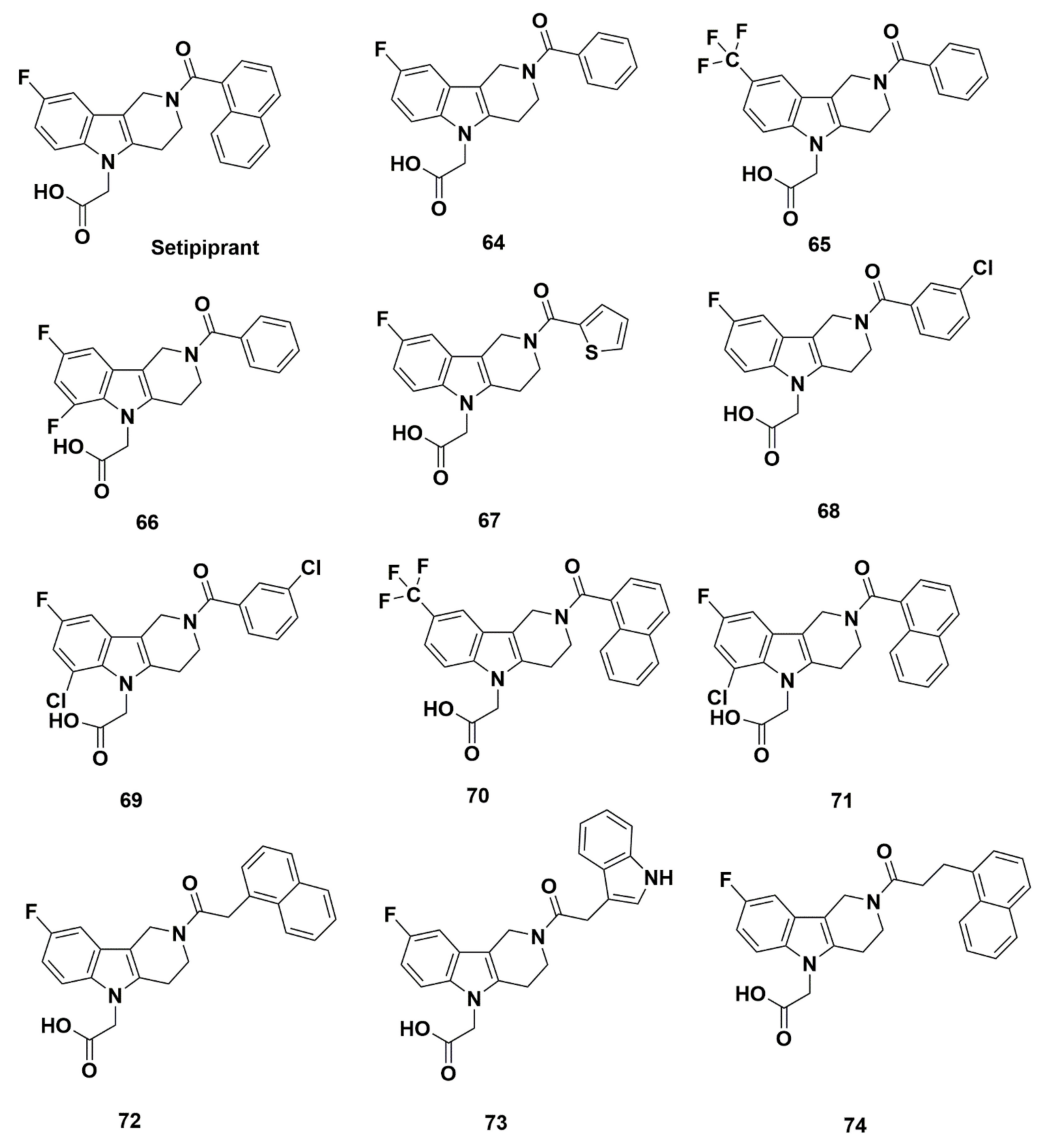
Molecular structures of **setipiprant** and compounds **64**–**74**.

In the same study as **setipiprant**, Fretz et al. characterized a number of other compounds (Figure 15) [77]. 2-(3,4-dihydro-1*H*-pyrido[4,3-b]indol-5(2*H*)-yl)acetic acid analogues were characterized for their affinity for GPR44. Fluorine-containing compounds **64** and **65** exhibited adequate IC_50_ values at 7 and 9 nM, respectively, measured in the presence of assay buffer solution. Additional characterization of compound **65** elucidated an IC_50_ value of 60 nM in binding in the presence of human serum albumin (HSA) and, an f_HSA_ value of 6. Additionally, IC_50_ values of 50 nM, 150 nM, and 130 nM were found through a cell based Ca^2+^ flux assay, cAMP homogeneous time resolved fluorescence (HTRF) assay, and human eosinophil shape change assay, respectively. Regarding the selectivity for prostanoid receptors hDP_1_, hEP_2_, and hEP_4_, IC_50_ values were greater than 10,000 for each of hDP_1_, hEP_2_ β-arrestin, and hEP_4_ β-arrestin, indicating negligible off-target binding. Additionally, the f**_Sel_** value was greater than 1100. Another fluorine-containing analogue, compound **66**, showed a less favorable IC_50_ of 35 nM (Figure 15).

Extensive changes to the precursor compound were made to produce the synthesized compounds (Figure 15). Out of compounds **67**–**74**, only compound **73** consistently produced IC_50_ values below 50 nM at 15 nM and 40 nM in two assays for Ca^2+^ flux and cAMP, respectively. The IC_50_ values for the other compounds **67**–**72** and **74** were determined using measurements of Ca^2+^ flux (40, 40, 1280, 20, 660, 70, and 65 nM, respectively) and cAMP (70, 95, not determined (nd), 380, nd, 160, and 160 nM, respectively). Additional binding studies in buffer (4, 9, 190, 11, 70, 4, 5, and 10 nM, respectively) and HSA (12, 60, 180, 70, 530, 180, 35, and 30 nM, respectively) for IC_50_ were also characterized (Table 5). The lower affinity of compounds **69** and **71** may be attributed to the substitution of chlorine and fluorine at the C6 and C8 position of the core, respectively. Additionally, **setipiprant** exhibited a substantially higher albumin shift up to a factor f_HSA_ of 57; this appears to be associated with the presence of a naphthyl or a naphthylmethyl moiety. In addition, all antagonists inhibited the PGD_2_ induced shape change of human eosinophils in the hESC assay, and compounds **67, 68** and **73** were discovered to antagonize hESC most effectively with IC_50_ < 100 nM. All compounds reduced COX-1 (prostaglandin-endoperoxide synthase) enzyme activity by less than 30% at 10,000 nM compound concentration (all IC_50_ values were determined well above 10,000 nM).

Selectivity of compounds **67**–**74** was further determined in a screen against DP_1_ (IC_50_ > 10,000, > 10,000, 4100, 2200, 310, > 10,000, > 10,000, > 10,000 nM, f_Sel_ > 2500, > 1100, 22, 200, 4.1, > 2500, > 2000, and > 10,000, respectively), EP_1_ (IC_50_ > 25,000 nM for all in Ca^2+^ assays), EP_2_ (IC_50_ > 10,000, > 10,000, > 10,000, > 10,000, 2700, > 10,000, > 10,000, and 9500 nM, respectively in the β-arrestin and binding assays), EP_3_ (IC_50_ > 25,000 nM for all in Ca^2+^ assays), EP_4_ (IC_50_ > 10,000 nM for all in the β-arrestin and binding assays), and TP_2_ (IC_50_ > 25,000 nM for all). Of note is that FLIPR assays against mouse, rat, guinea pig, and dog GPR44 revealed that all compounds exhibited antagonistic effects when binding to GPR44 with comparable potencies, except for compound **68**, which were identified as partial agonists on the mouse and the guinea pig receptor. This indicates that the choice of species used to develop PET tracers from compound **68** will have to be carefully considered. The physicochemical properties of the compounds with f_Sel_ > 200 in favor of hCRTH_2_ were further studied. The Cl_int_ values of compounds **67** and **68** in rat liver microsomes were low at 8 and less than 4 μL/min/mg protein, respectively. However, the Cl_int_ values of compounds **72** and **73** were high, indicating they may be prone to oxidative metabolism. The values of compounds **72** and **73** were between 23 and 63 μL/min/mg protein. However, none of the Cl_int_ values in rat hepatocytes were above 5.3 μL/min/10^6^ cells, indicating low clearance in the liver. The plasma clearance (measured as Cl) for compound **67** was found to be detrimentally high (36 mL/min/kg). For compound **68**, the low plasma clearance (5.9 mL/min/kg) and moderate exposure did not exempt it from a low oral bioavailability of 23%. Meanwhile, the exposure for compound **73** was dramatically low at 62 ng/h/mL and led to a bioavailability of 2%. Additional pharmacokinetic properties were elucidated from male beagle dogs orally administered 10 mg/kg and intravenously administered 1 mg/kg of compound **68** and **setipiprant**. The results show that the exposure was high at 9620 and 91,100 ng h^−1^ mL^−1^, respectively, and bioavailability was adequate at 64% and 55%, respectively. However, compound **68** demonstrated a greater clearance of 11 mL min^−1^ kg^−1^.

##### Recommendations

**Setipiprant** is an encouraging candidate for development of PET tracers to characterize BCM, particularly because it has been highly characterized as a potent compound both in vitro and in vivo. Among the analogues of **setipiprant**, compounds **64** and **65** presented favorable IC_50_ values, although additional characterizations will have to be made. It is worth noting that radiolabeling a trifluoromethyl group, such as those contained in compound **65**, has been traditionally difficult due to isotopic dilution with fluorine-19. However, methods including using a novel radiofluorination reagent and reduction of base-cryptand concentration have been developed, through which one fluorine-18 is introduced into the molecule [85]. Other compounds may be used as a reference in developing new PET tracers, such as compounds **72** and **74**, which exhibited excellent to moderate IC_50_ values, specificity for GPR44, preferable plasma clearance, and favorable bioavailability.

#### 2.2.6. CT-133 (Compound **45**)

**Figure 16:**
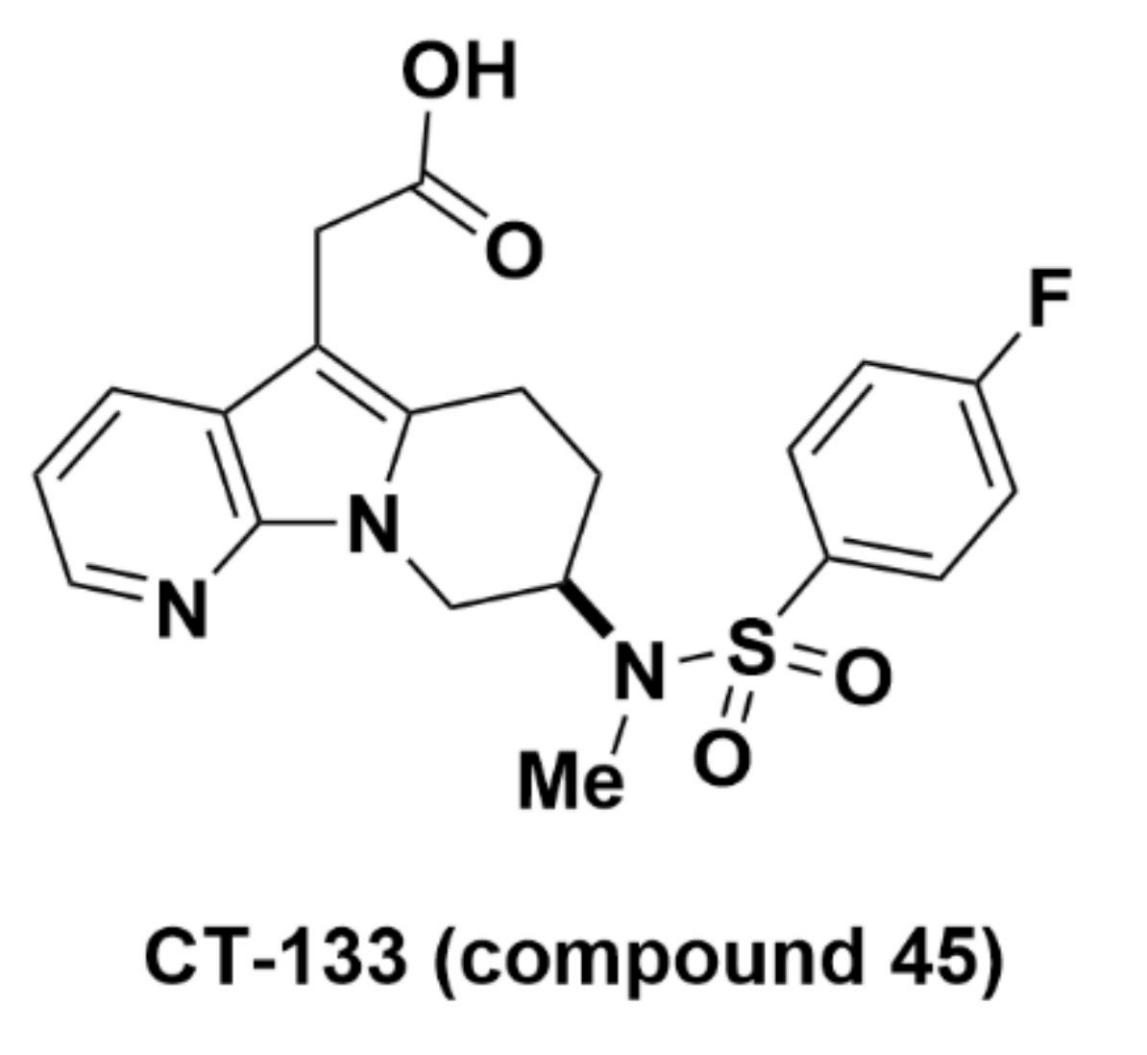
Molecular structure of **CT-133** (compound **45**).

**CT-133** (namely, compound **45**; {8-[(4-fluoro-benzenesulfonyl)-methyl-amino]-6,7,8,9-tetrahydro-pyrido[3,2-b]indolizin-5-yl}-sodium acetic acid) was further evaluated by Guo et al. at the CSPC Pharmaceutical Group in 2015 (Figure 16) [86]. The pharmacokinetics of **CT-133** was also promising in vivo, with satisfactory bioavailability. In mouse, dog, and rat, oral administration of **CT-133** showed bioavailability of 89.6%, 85.4%, and 71.6%, respectively, with little difference in repeated and single administration in rat. The safety profile was determined to be favorable, and there was no induction of metabolic enzymes. In another example of **CT-133**’s safety, rat dosed with up to 2000 mg/kg/day and dog dosed with up to 1000 mg/kg/day or 100 mg/kg/day for seven days showed no concerning reaction. Later, **CT-133** was shown to be a potent antagonist for treatment of disease. In lipopolysaccharide-induced acute lung injury, 10 or 30 mg/kg **CT-133** administered intragastrically suppressed neutrophil and macrophage cell count dose-dependently and migration to PGD_2_ locations in vitro and in mouse lungs [87,88].

In silico analysis provided further details into the mechanisms behind optimized binding of **CT-133** to GPR44. **CT-133** binds to a partially occluded region on extracellular GPR44 enclosed by the ligand entrance. Comparison of PGD_2_ and **CT-133** binding on the region by estimates of the root mean square deviation (RMSD), root mean square fluctuation (RMSF), and surface-exposure using the solvent accessible surface area (SASA) showed values of 1.80 Å (PGD_2_), 1.44 Å (**CT-133**), and 1.75 Å (unbound); 2.32 Å (PGD_2_), 2.17 Å (**CT-133**), and 3.10 Å (unbound); and 245805 Å^2^ (PGD_2_) and 187765 Å^2^ (**CT-133**), respectively. **CT-133**’s low RMSD value suggests that the compound significantly stabilizes the binding region, perhaps favoring **CT-133** binding over PGD_2_. The stabilization is corroborated by the low RMSF and SASA values, representing the residues’ motion restriction and reduced orientations, respectively. In the active site, PGD_2_ and **CT-133** comparison yielded RMSD values of 1.52 Å and 0.68 Å, respectively, again showing the higher stability and binding affinity of **CT-133**. Inhibitor qualities of **CT-133** were confirmed by RMSD values (2.74 Å) of binding to helix 8, a region believed to assist inhibition of GPR44 function, which were lower than that for PGD_2_ (3.55 Å) or the unbound receptor (3.426 Å). Higher RMSF values of 4.53 Å for **CT-133** relative to relative to 3.54 Å for PGD_2_ represented greater **CT-133**-mediated helix 8 flexibility. The RMSD and RMSF of **CT-133** binding to helix 8 both represent more optimal inhibitor qualities.

The binding affinity for GPR44, estimated through a MM/PBSA-based approach, showed total free energy values of -67.50 kcal/mol for **CT-133** and -50.12 kcal/mol for PGD_2_. The lower binding energy has been attributed to the 7-azaindole group that causes **CT-133** to bind more favorably to GPR44 than PGD_2_ [89].

##### Recommendations

Due to its strong binding affinity and selectivity for GPR44 (see Section 2.2.3. Merck Analogues), in addition to confirmed pharmacokinetics in vivo, **CT-133** is a promising compound for use in development of GPR44-specific PET tracers.

### 2.3. Radiosynthesis Strategies for ^18^F-Labeled Ramatroban-Based Analogues

The 77 selected **ramatroban** analogues containing a fluorine nuclide were characterized for properties including binding affinity, off-target binding, and pharmacokinetic and metabolic profile. Among these analogues, 32 compounds with favorable properties were recommended as potential ^18^F-labeled GPR44 PET tracers. Late-stage radiofluorination and the building block approach are two major strategies for radiosynthesis of ^18^F-labeled tracers. In recent years, novel and promising methodologies to implement the two strategies of aromatic radiofluorination have been developed and discussed in the comprehensive reviews by Preshlock et al., Brooks et al., and van der Born et al. [90,91,92]. Additionally, strategies are being actively developed and reported, which enable ^18^F-labeling in places that are traditionally challenging. For example, Chen et al. described a method for direct ^18^F-labeling [93]. Given these strategies, it would be beneficial to successfully generate the recommended potential ^18^F-labeled GPR44 analogues.

**Scheme 1:**
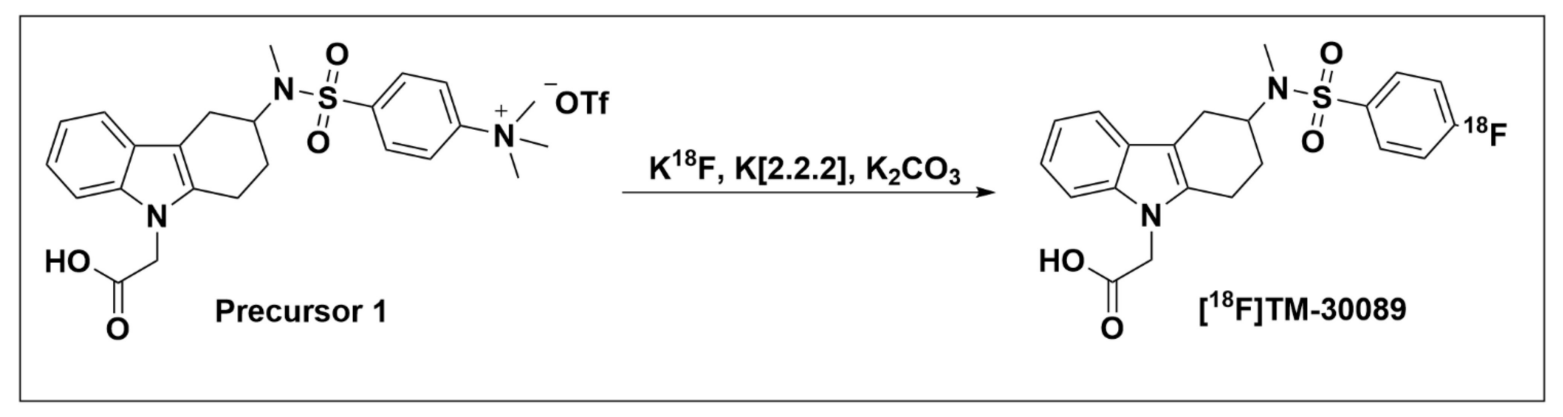
Radiosynthesis of **[^18^F]-TM-30089**.

Our lab recently followed the strategy outlined above in selecting promising compounds to develop GPR44-specific PET radiotracers and utilized nucleophilic substitution to radiolabel with fluorine-18 a precursor (Precursor **1**, 4-(*N*-(9-(carboxymethyl)-2,3,4,9-tetrahydro-1*H*-carbazol-3-yl)-*N*-methylsulfamoyl)-*N,N,N*-trimethylbenzenaminium-trifluoromethanesulfonate) of the selective GPR44 antagonist, **TM-30089** (K_i_ = 0.6 nM) (Scheme 1). The resulting novel GPR44 tracer, **[^18^F]-TM-30089** ([^18^F]-({3-[(4-fluoro-benzenesulfonyl)methyl-amino]-1,2,3,4-tetrahydro-carbazol-9-yl}acetic acid), demonstrated high radiochemical yield (30%) and radiochemical purity (> 98%). **[^18^F]-TM-30089** biodistribution analysis additionally showed low tracer uptake in NOD/SCID mouse pancreas, matching the characteristic GPR44 expression present there. The manuscript is currently being prepared for publication.

## 3. Conclusions

Taken together, the existing pool of **ramatroban** analogues containing fluorine groups could serve as a source of promising GPR44 ^18^F-labeled PET tracers to investigate metabolic diseases longitudinally, monitor beta cell therapies, and evaluate pharmaceutical drug efficacy. To develop these compounds into PET tracers, direct and indirect radiolabeling methods may be utilized to incorporate fluorine-18. Our group followed the outlined approach and effectively developed **[^18^F]-TM-30089** in an unpublished study. Therefore, there is great potential for future development of ^18^F-labeled PET tracers targeting GPR44 to accurately monitor and quantify BCM.

## Figures and Tables

**Table 1:** Binding affinity and functional antagonism on hCRTH_2_ for preliminary aryl amides and structure–activity relationship (SAR) on the amide linker (values were obtained from [73]).

Compd.	hCRTH_2_^a^ (K_i_)	cAMP ^b^ (IC_50_)	EOS ^c,d^ (IC_50_)
**27**	340 nM	2500 nM	
**28**	7.4 nM	12 nM	4.8 nM
**29**	3.0 nM	6.2 nM	6.0 nM
**30**	2.0 nM	2.0 nM	4.4 nM
**31**	1.8 nM	4.0 nM	
**32**	1.7 nM	4.0 nM	1.4 nM
**33**	4.3 nM	8.0 nM	2.4 nM
**34**	141 nM		
**35**	70 nM		
**36**	22 nM		
**37**	126 nM		
**38**	19 nM		
**39**	408 nM		
**40**	4.0 nM	3.0 nM	0.96 nM
**41**	39 nM	30 nM	

^a^ Radioligand competition binding assay using membrane proteins from HEK293 (EBNA) cells stably expressing the receptor CRTH_2_ in a 10 mM solution of HEPES/KOH (all values are mean of two or more experiments). ^b^ Functional assay: the intracellular concentration of cAMP was determined using the ^125^I-cAMP scintillation proximity assay. The assay was performed in Hank’s balanced salt solution 25 mM HEPES containing 5000 nM Forskolin (K_i_ is an average of at least two independent titrations). ^c^ IC_50_s are an average of at least two independent titrations. ^d^ Whole blood eosinophil shape change assay.

**Table 2:** Activities of azaindole sulfonamides and amides (values were obtained from [74]).

Compd.	hCRTH_2_^a^ (K_i_)	*c*AMP ^b^ (IC_50_)	EOS ^c^ (IC_50_)	DP ^a^ (K_i_)	TP ^a^ (K_i_)
**42**	7189 nM			> 1300 nM	> 7000 nM
**43**	6725 nM			> 3400 nM	> 6800 nM
**44**	139 nM	351 nM		> 12,000 nM	> 22,000 nM
**45**	3.3 nM	3.4 nM	7.0 nM	> 47,000 nM	> 22,000 nM
**46**	1.8 nM	3.2 nM	3.3 nM	> 18,000 nM	> 27,000 nM
**47**	1.9 nM	3.6 nM	15.8 nM	> 3800 nM	> 1000 nM
**48**	3.4 nM	5.7 nM	1.2 nM	> 38,000 nM	> 71,000 nM
**49**	21.1 nM	73.8 nM		> 12,000 nM	> 22,000 nM
**50**	4.7 nM	3.5 nM	2.3 nM	> 10,000 nM	> 21,000 nM
**51**	3.6 nM	7.5 nM	3.1 nM	> 3700 nM	> 6800 nM
**52** ^d^	5.1 nM	4.4 nM	2.4 nM	> 4000 nM	> 7200 nM
**53**	3.4 nM	4.7 nM	1.2 nM	> 4000 nM	> 7200 nM
**54**	3.9 nM	4.5 nM	3.4 nM	> 4000 nM	> 7200 nM
**55**	11.5 nM	7.0 nM		> 4000 nM	> 7200 nM

^a^ Radioligand competition binding assay using membrane proteins from HEK293 (EBNA) cells stably expressing the receptor hCRTH_2_ in a 10 mM solution of HEPES/KOH (all values are mean of two or more experiments). ^b^ Functional assay: the intracellular concentration of cAMP was determined using the ^125^I-cAMP scintillation proximity assay. The assay was performed in Hank’s balanced salt solution 25 mM HEPES containing 5000 nM Forskolin (all values are mean of two or more experiments). ^c^ Human whole blood eosinophil shape change assay (all values are mean of two or more experiments). ^d^ Diastereomeric mixture.

**Table 3:** Preliminary SAR of compounds **57**–**61** (values were obtained from [75]).

Compd.	CRTH_2_ Binding (IC_50_^a^)	Log D_7.4_	Rat Hep Cl_int_^b^	Hum Mic Cl_int_^c^	Agonism EOS Shape Change ^d^	CRTH_2_ Ca^2+^ (IC_50_^e^)
**57**	18 nM	0.4	<4	6	IA	517 nM
**58**	316 nM		<3	5		
**59**	10 nM	1.0	<3	11	IA	
**60**	1.0 nM		<3	<3	IA	9 nM
**61**	0.3 nM	0.8	<3	<3	yes	

^a^ Radiometric binding assay, *n* > 2 measurements. ^b^ Rat hepatocyte intrinsic clearance (µL/min/1 × 10^6^ cells). ^c^ Human liver microsomes intrinsic clearance (µL/min/mg). ^d^ Agonism in eosinophil shape change, IA refers to <10% response of DK-PGD_2_. ^e^ Antagonism of PGD_2_-mediated Ca^2+^ flux in human CHO cells, *n* > 2 measurements.

**Table 4:** Plasma pharmacokinetic variables of **setipiprant** in healthy subjects after administration of a single 500 mg dose of **setipiprant** as a capsule (*n* = 20) or tablet (*n* = 20) (values were obtained from [79]).

Variable	Capsule ^a^	Tablet ^a^	Tablet: Capsule ^b^
C_max_, µg/mL	6.44 (5.46–7.58)	6.04 (4.72–7.74)	0.94 (0.79–1.12)
t_max_, h	3.00 (1.50–5.00)	3.50 (1.00–5.00)	0.00 (−0.50–1.00)
t_1/2_, h	11.12 (9.76–12.67)	11.40 (10.54–12.34)	1.03 (0.93–1.13)
AUC_0−∞_, µg∙h/mL	31.50 (26.52–37.40)	31.88 (26.54–38.31)	1.01 (0.92–1.12)

^a^ Values indicate geometric means (95% CIs), except for t_max_, for which median and range are presented. ^b^ Values indicate ratio of geometric means (90% CIs), except for t_max_, for which difference of medians (90% CIs) is presented.

**Table 5:** SAR study exploring compounds **65** and **67**–**74**, with IC_50_ binding data for hCRTH_2_ of selected compounds, measured in the presence and absence of human serum albumin (HSA) in the assay buffer^a^ (values were obtained from [77]).

Compd.	hCRTH_2_ Receptor Interaction						Prostanoid Receptor Interaction			
	Binding in			Ca^2+^ Flux ^a^	cAMP	hESC	hDP_1_	f_Sel_^c^	hEP_2_	hEP_4_
	Buffer IC_50_^d^	HSA IC_50_^d^	f_HSA_^b^	IC_50_^d^	IC_50_^d^	IC_50_^d^	Binding IC_50_^d^		β-Arrestin IC_50_^d^	β-Arrestin IC_50_^d^
**Setipiprant**	6	340	57	30	80	235	1290	215	2600	>10,000
**65**	9	60	6	50	160	130	>10,000	>1100	>10,000	>10,000
**67**	4	12	3	40	70	34	>10,000	>2500	>10,000	>10,000
**68**	9	60	7	40	95	60	>10,000	>1100	>10,000	>10,000
**69**	190	180	1	1280	nd	nd	4100	22	>10,000	>10,000
**70**	11	70	6	20	380	180	2200	200	>10,000	>10,000
**71**	70	530	7	660	nd	nd	310	4.1	2700	>10,000
**72**	4	180	45	70	160	275	>10,000	>2500	>10,000	>10,000
**73**	5	35	7	15	40	50	>10,000	>2000	>10,000	>10,000
**74**	10	30	3	65	160	210	>10,000	>1000	9500	>10,000

nd = not determined.^a^ IC_50_ values are given for the effects in the cell based Ca^2+^ flux assay, the cAMP homogeneous time resolved fluorescence (HTRF) assay, and the human eosinophil shape change assay. Data are given to demonstrate selectivity against prostanoid receptors hDP_1_, hEP_1-4_, and hTP_2_. The IC_50_ values represent the mean from at least three independent experiments if not stated otherwise. ^b^ Human serum albumin shift factor f_HSA_ = IC_50_ (HSA)/IC_50_ (buffer). ^c^ Selectivity factor f_Sel_ = IC_50_ (hDP_1_)/IC_50_ (hCRTH_2_). ^d^ Units are nM.
